# Frontal White Matter Anisotropy and Antidepressant Remission in Late-Life Depression

**DOI:** 10.1371/journal.pone.0003267

**Published:** 2008-09-24

**Authors:** Warren D. Taylor, Maragatha Kuchibhatla, Martha E. Payne, James R. MacFall, Yvette I. Sheline, K. Ranga Krishnan, P. Murali Doraiswamy

**Affiliations:** 1 The Department of Biostatistics and Bioinformatics, Durham, North Carolina, United States of America; 2 The Department of Psychiatry, Durham, North Carolina, United States of America; 3 The Department of Radiology, Durham, North Carolina, United States of America; 4 The Center for the Study of Aging and Human Development, Durham, North Carolina, United States of America; 5 The Neuropsychiatric Imaging Research Laboratory, Durham, North Carolina, United States of America; 6 The Department of Psychiatry, Washington University, St. Louis, Missouri, United States of America; 7 The Duke-NUS Graduate Medical School Singapore, Singapore, Singapore; University of Granada, Spain

## Abstract

**Introduction:**

Neuroanatomic features associated with antidepressant treatment outcomes in older depressed individuals are not well established. This study used diffusion tensor imaging to examine frontal white matter structure in depressed subjects undergoing a 12-week trial of sertraline. We hypothesized that remission would be associated with higher frontal anisotropy measures, and failure to remit with lower anisotropy.

**Methods:**

74 subjects with Major Depressive Disorder and age 60 years or older were enrolled in a twelve-week open-label trial of sertraline and completed clinical assessments and 1.5T magnetic resonance brain imaging. The apparent diffusion coefficient (ADC) and fractional anisotropy (FA) were measured in regions of interest placed in the white matter of the dorsolateral prefrontal cortex, anterior cingulate cortex, and corpus callosum. Differences in ADC and FA values between subjects who did and did not remit to treatment over the study period were assessed using generalized estimating equations, controlling for age, sex, medical comorbidity and baseline depression severity.

**Results:**

Subjects who did not remit to sertraline exhibited higher FA values in the superior frontal gyri and anterior cingulate cortices bilaterally. There were no statistically significant associations between ADC measures and remission.

**Conclusions:**

Failure to remit to sertraline is associated with higher frontal FA values. Functional imaging studies demonstrate that depression is characterized by functional disconnection between frontal and limbic regions. Those individuals where this disconnection is related to structural changes as detected by DTI may be more likely to respond to antidepressants.

**Trial Registration:**

ClinicalTrials.gov NCT00339066

## Introduction

Depression in older populations can differ from depression in younger adult individuals and is often associated with chronic medical illness, disability, and cognitive impairment. It is also associated with aging-related brain changes including the presence of hyperintense lesions, bright areas occurring in the brain parenchyma as seen on T2-weighted magnetic resonance imaging (MRI). These changes are primarily ischemic in origin [1], and although are observed in normal aging, are often more severe in older depressed individuals [2].

The relationship between these hyperintense lesions and antidepressant treatment outcomes is unclear. Several studies have concluded that greater hyperintense lesion severity is associated with poorer response to antidepressants [3]–[7]. Greater hyperintensity severity is additionally associated with significantly more adverse drug reactions [8], which may result in early drug discontinuation or inability to increase doses to therapeutic levels. In contrast, other studies have not found a relationship between cross-sectional lesion severity and acute antidepressant outcomes [9]–[16], although relationships may exist between longitudinal change in lesion severity and longer-term course of depression [11].

More recent work investigating late-life depression has utilized diffusion tensor imaging (DTI). DTI can quantify water diffusion, which in living tissue is constrained by neuronal integrity and modulated by myelin [17]. DTI measures include the apparent diffusion coefficient (ADC), a general measure of diffusion which may serve as a surrogate marker for fiber density [18], and anisotropy, which measures the direction of water diffusion and has been proposed to be a surrogate marker for white matter orientation and organization. Similar to what is observed in old stroke regions, white matter hyperintense lesions increase ADC and decrease anisotropy [19], and greater hyperintense lesion severity is associated with more widespread alterations in DTI measures even in normal appearing white matter [20]. Although several studies have reported that depressed elders exhibit reduced frontal and temporal anisotropy [21]–[24], this technique has not been used as extensively to study treatment response. One group has examined the relationship between DTI measures and antidepressant response in geriatric depression, associating failure to achieve remission with reduced fractional anisotropy in multiple regions, including the cingulate gyrus and dorsolateral prefrontal cortex [25], [26].

The purpose of this study was to use DTI to examine if measures of frontal white matter microstructure were associated with acute 12-week response to sertraline. We hypothesized that lower fractional anisotropy (FA) and higher diffusivity (measured as ADC), a pattern of findings associated with both chronic stroke and hyperintense lesions [19], [20], would be associated with failure to achieve remission. In turn, we hypothesized that individuals who remitted would exhibit higher frontal FA measures.

## Methods

The protocol for this trial and supporting CONSORT checklist are available as supporting information; see Checklist S1 and Protocol S1.

### Sample

Subjects were recruited from advertisements and outpatient clinical referrals at Duke University Medical Center. To be eligible, subjects had to be age 60 years or older, meet DSM-IV criteria for Major Depressive Disorder without psychosis as assessed through clinical evaluation and the Structured Clinical Interview for DSM-IV (SCID) [27], and exhibit a baseline Montgomery-Asberg Depression Rating Scale [28] score of 18 or greater. Exclusion criteria included: 1) any MRI contraindications; 2) comorbid Axis I diagnosis, including current substance abuse or bipolar disorder; 3) active suicidality; 4) current psychotic symptoms, 5) severe or unstable medical conditions; 6) primary neurological disorders including dementia or stroke; 7) Mini-Mental State Examination (MMSE) [29] score<24; 8) current depressive episode that failed to respond to adequate trials of two prior antidepressants administered for at least 6 weeks at therapeutic doses, or had failed an adequate trial of sertraline; 9) current psychotherapy.

All study procedures were explained to each participant, and those who provided written informed consent were enrolled. The study was approved by the Duke University Health System Institutional Review Board.

### Study Design and Clinical Measures

Baseline data acquisition included assessing demographic data, depression severity using the MADRS, and severity of medical burden using the Cumulative Illness Rating Scale (CIRS) modified for geriatric populations [30]. Age of depression onset was ascertained through the SCID and review of medical records.

After screening, subjects taking antidepressant medications at doses approved to treat depression underwent a washout period of up to two weeks, while those taking lower doses had the antidepressant stopped without a washout. Eleven subjects had antidepressants discontinued after enrollment, while two had St. John's Wort discontinued. The study protocol allowed limited use of zolpidem or zaleplon for sleep, or lorazepam for anxiety. Subjects on other concomitant medications for medical indications, sleep, or anxiety could continue them provided doses remained stable. Eleven subjects required hypnotics for sleep, seven required benzodiazepines for anxiety, and seven subjects were on anticonvulsants or benzodiazepines for medical indications such as neuropathy or restless legs syndrome.

After completing any needed washout, subjects received sertraline for twelve weeks. They were generally started at 25 mg for a few days to rule out drug sensitivity, and then increased to 50 mg daily. Subjects were assessed at 2, 4, 6, 8, and 12 weeks. A dose increase of 50 mg was allowed at each visit, up to a possible maximum dose of 200 mg daily, and the decision to titrate the dose was guided by the Clinical Global Impression – Severity scale (CGI-S) [31]. Subjects with a CGI-S of 3 or greater had a dose increase unless there was a concern for tolerability. Subjects with a CGI-S of 2 could be titrated at investigator discretion, and a CGI-S of 1 was considered to be in remission. If a dose increase resulted in intolerable side effects, the dose could be reduced to the previously tolerated level. Depression severity was assessed at each visit using the MADRS.

### MRI Acquisition and Processing

Subjects were imaged with a 1.5 T whole-body, research-dedicated MRI (Signa, GE Healthcare) using a standard head (volumetric) radiofrequency coil. The scanner alignment light was used to adjust head tilt and rotation so the axial plane light passed across the canthomeatal line and the sagittal lines were aligned with the center of the nose. A rapid sagittal localizer scan was acquired to confirm alignment. MRI was obtained within the first two weeks of study participation, typically at the first follow-up visit after initiating study drug.

The diffusion tensor images were acquired using a single shot 2D diffusion tensor echo planar pulse sequence in the axial plane with a 32 cm field-of-view, 3 mm slice thickness (no gaps between slices), relaxation time (Tr) = 6000, excitation time (Te) = 100, 128 (phase)×128 (frequency), full imaging bandwidth = 180 KHz, 4 signal averages, 6 diffusion directions, each with a b-value of 1000 sec/mm^2^ plus an acquisition with b = 0 using the Basser scheme [32].

Diffusion tensor images were processed using custom programs run on MATLAB software (version 7, The MathWorks, Natick, MA) that calculated the diffusion tensor eigenvalues in each voxel [33]. FA and ADC images were calculated, and regions-of-interest (ROIs) were placed by a single analyst (WDT) using Analyze software (version 6.5, Mayo Clinic). Scans were coded with no identifiable information, and the analyst was blinded to subject identity and treatment outcome.

Oval ROIs of identical size (45.7 mm^2^) were used to measure FA and ADC values. The same ROI was used for both measures, and all ROIs were placed on axial slices. Hyperintense lesions were avoided for all ROI placements. This decision was made based on the observation that hyperintensities can have strong effects on DTI measures that is not representative of the DTI measures of surrounding white matter [19]. There were no cases where the presence of hyperintense lesions prevented appropriate ROI placement according to ROI placement procedures.

ROI placement procedures have been previously described [20], [21]. The superior (SFG) and middle frontal gyri (MFG) ROIs were placed halfway between the precentral sulcus and anterior boundary of the brain, on the most inferior slice where both gyri were visible as separate structures. The anterior cingulate cortex (ACC) ROIs were placed in the white matter lateral to the cingulated gyrus, on the most inferior slice where the anterior horns of the lateral ventricle were still visible. For the corpus callosum, two ROIs were placed to either side of midline, on the slice ventral to the slice where it was divided by the longitudinal fissure. Results from these two separate corpus callosum ROIs were averaged together to create a composite measure. We used the corpus callosum as a control region that we did not expect to be associated with antidepressant outcomes as it is a region of high anisotropy that was not different between depressed and nondepressed elders in previous studies [21].

Reliability was established by repeated measurements on multiple DTI scans. Intraclass correlation coefficients (ICCs) of FA measures were: left ACC, 0.910; right ACC, 0.992; left SFG, 0.991; right SFG, 0.985; left MFG, 0.975; right MFG, 0.983; left corpus callosum, 0.971; right corpus callosum, 0.934. ICCs for ADC measures (using the same ROIs as the FA measures) were: left ACC, 0.945; right ACC, 0.957; left SFG, 0.978; right SFG, 0.987; left MFG, 0.931; right MFG, 0.893; left corpus callosum, 0.751; right corpus callosum, 0.799.

### Statistical Analysis

SAS software (version 9.1, SAS Institute, Cary, NC) was used for all statistical analyses. Remission was defined as achieving a MADRS of 10 or less at any assessment, as this cutoff has been shown to provide a valid definition [34]. Bivariate analyses of demographic and neuroimaging data were conducted between remitted and nonremitted groups, using the chi-square test for categorical variables and two-tailed t-test for continuous variables, or the Satterthwaite t-test if variances were not equal. Normality of neuroimaging data was assessed both graphically and by Wilks test.

Primary analyses used the GENMOD procedure in SAS to develop generalized estimating equation (GEE) models [35] using an exchangeable correlation structure which examined the relationship between the repeated outcome measure of failure to remit at each assessment point and the independent variables. For missing data, the GEE models assume missing completely at random (MCAR). The dependent measure was the repeated measure dichotomous variable of remitted or not remitted, and we modeled the probability of failing to remit. Independent variables included the DTI measure, baseline depression severity measured with the MADRS, age, sex, medical comorbidity measured using the CIRS, and a variable accounting for which assessment period was being evaluated (baseline, 2-, 4-, 6-, 8- or 12-week). These covariates were selected for the model as they were either significantly different between the remitting and non-remitting groups (age, baseline MADRS) or have been previously associated with antidepressant outcomes in late-life depression (medical comorbidity, [36]; sex, [37]). In an exploratory step, we also tested for a DTI measure – age interaction and a DTI measure – baseline MADRS interaction effect on remission. Multicolinearity between covariates was assessed in models by examining variance inflation factors (VIF); all VIFs were less than 30. Separate models were created to examine each DTI measure. We did not control for multiple comparisons.

In secondary analyses, we sought to examine if DTI measures were associated with time to remission, an approach used by others [25]. This survival model used the PHREG procedures in SAS [38], examining the time to first remission or last assessment, at which point subjects were censored. Independent variables included DTI measures, age, baseline MADRS score, sex, and CIRS score.

## Results

**Figure 1:**
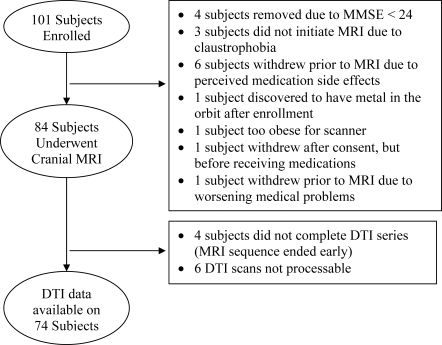
Subject flow. Details of subject enrollment and why excluded from the present study.

101 depressed subjects signed consent and enrolled in the study between January 2002 and March 2006; 74 of those subjects are included in this analysis (Figure 1). 35 subjects were female and 39 were male, with a mean age of 68.1 years (range 60–95 years, standard deviation = SD = 7.3 years). The group's mean initial MADRS score was 25.4 (SD = 4.2, range 18–37); at study completion, the mean final MADRS score was 11.5 (SD = 7.9, range 0–42), with a mean sertraline daily dose of 102.0 mg (SD = 37.6 mg, range 25 mg–200 mg).

Over the course of the twelve-week study, 37 subjects achieved a MADRS score of 10 or less and achieved remission, while 37 subjects did not. 67 subjects completed all 12 weeks of the study. Four subjects completed only 4 weeks, 1 completed only 6 weeks, and 2 completed only 8 weeks. All of the subjects who withdrew early were classified as not achieving remission, except one of the subjects who completed only 8 weeks who did remit. Remitted subjects were younger and less severely depressed at baseline (Table 1). We next tested for bivariate differences in unadjusted FA and ADC measures between subjects who did and did not remit (Table 2). In these analyses, the only measure significantly different between remitted and nonremitted groups was the FA value of the right anterior cingulate cortex.

**Table 1:** Demographic characteristics between subjects who did and did not remit.

	Remitted (N = 37)	Nonremitted (N = 37)	Test Result	*df*	*p* value
Age	65.8 (5.7)	70.5 (8.0)	t = 2.94	64.9	0.0045
Sex (% Female)	43.2% (16/37)	48.6% (18/37)	χ^2^ = 0.49	1	0.4849
Race (% Caucasian)	94.6% (35/37)	94.6% (35/37)	-	1	1.0000
Age of Onset	55.0 (15.8)	55.0 (16.4)	t = 0.00	72	1.0000
CIRS	6.5 (2.8)	7.4 (3.6)	t = 1.26	72	0.2133
MMSE	28.3 (1.7)	27.6 (1.7)	t = 1.74	72	0.0857
MADRS, baseline	24.4 (3.8)	26.4 (4.5)	t = 2.05	72	0.0438
MADRS, final	5.4 (2.9)	17.6 (6.4)	t = 10.51	50.3	<0.0001
Final Sertraline Dose	97.8 (36.5)	106.1 (38.8)	t = 0.93	72	0.3578

*Age and age of onset presented in years. Final of sertraline is in milligrams. Continuous variables presented as mean (standard deviation). Two-tailed pooled t-tests used for continuous variables (Satterthwaite t-test for unequal variances), chi-square test for sex, and Fisher's exact test for race. CIRS = Cumulative Illness Rating Scale; MADRS = Montgomery-Asberg Depression Rating Scale.*

**Table 2:** Unadjusted differences in DTI measures between subjects who did and did not remit.

	Remitted (N = 37)	Nonremitted (N = 37)	t value	*df*	*p* value
FA values					
• SFG, left	0.408 (0.080)	0.421 (0.082)	0.80	72	0.4284
• SFG, right	0.392 (0.069)	0.402 (0.080)	0.53	72	0.6007
• MFG, left	0.283 (0.064)	0.262 (0.046)	1.54	72	0.1284
• MFG, right	0.268 (0.052)	0.264 (0.049)	0.34	72	0.7351
• ACC, left	0.365 (0.063)	0.374 (0.076)	0.56	72	0.5802
• ACC, right	0.332 (0.055)	0.368 (0.072)	2.44	72	0.0170
• CC, genu	0.423 (0.070)	0.454 (0.101)	1.51	63.9	0.1343
ADC values					
• SFG, left	76.1 (5.8)	77.4 (11.2)	0.63	53.8	0.5308
• SFG, right	76.2 (5.2)	77.2 (11.4)	0.48	50.1	0.6344
• MFG, left	77.5 (7.3)	79.0 (6.1)	0.91	72	0.3643
• MFG, right	79.6 (6.2)	80.1 (5.8)	0.30	72	0.7636
• ACC, left	83.2 (5.8)	83.4 (5.0)	0.21	72	0.8348
• ACC, right	82.6 (4.9)	84.0 (6.9)	0.97	65	0.3342
• CC, genu	104.6 (14.4)	101.7 (15.4)	0.82	72	0.4122

*FA = fractional anisotropy, ADC = apparent diffusion coefficient, SFG = superior frontal gyrus, MFG = middle frontal gyrus, ACC = anterior cingulate cortex, CC = corpus callosum. All results presented as mean (standard deviation). All tests reported using two-tailed pooled t-tests, except when unequal variances were present, in which case a Satterthwaite t-test was used.*

Table 3 details the results of models examining the relationship between failure to remit and regional FA, while also controlling for baseline depression severity, age, sex, and medical comorbidity as measured with the CIRS. In these models, higher anisotropy of the white matter of the superior frontal gyri bilaterally and anterior cingulate cortices bilaterally was significantly associated with failure to remit. In these models where FA was significantly associated with lack of remission, higher baseline MADRS (*p*<0.01 all models) and greater age (*p*<0.05 all models) were also associated with nonremission, while neither gender nor CIRS score reached a level of statistical significance in any model. Similar models were created examining the relationship between ADC values and nonremission; however ADC was not associated with nonremission in any model (data not shown). When DTI-age and DTI-MADRS interactions were included, these terms did not reach statistical significance in any model.

**Table 3:** Relationship between FA values and remission in multivariate models.

Regional FA value	Estimate	SE	Test Result (Z-score)	*p* value
SFG, left	0.0099	0.0036	2.72	0.0064
SFG, right	0.0068	0.0030	2.25	0.0244
MFG, left	0.0007	0.0032	0.20	0.8405
MFG, right	0.0050	0.0043	1.16	0.2476
ACC, left	0.0079	0.0036	2.18	0.0290
ACC, right	0.0088	0.0036	2.42	0.0156
CC, genu	0.0035	0.0028	1.26	0.2080

*FA = fractional anisotropy; SFG = superior frontal gyrus; MFG = middle frontal gyrus; ACC = anterior cingulate cortex; CC = corpus callosum. Models examined remission of depression as the dependent variable, with FA, age, sex, baseline depression severity and medical comorbidity as independent variables.*

Finally, we used an approach similar to that found in a previous report [25] to determine if these DTI measures were associated with a time to first remission. The 37 subjects who remitted has a mean time to remission of 6.8 weeks (SD = 2.8 weeks, median = 6 weeks). In models controlling for baseline MADRS score, age, sex, and CIRS score, no DTI measure was significantly associated with time to remission (data not shown).

## Discussion

Contrary to our hypothesis, we found that higher FA measures, not lower, were associated with a failure to achieve remission. These measures were of the anterior cingulate and superior frontal gyrus, regions previously identified as exhibiting depression-related differences on DTI [21] and associated with antidepressant response in functional imaging studies [39]. These relationships were statistically significant after controlling for age, sex, baseline depression severity, and medical comorbidity. We found no association between ADC and remission.

These results are discrepant with the two other published studies examining the relationship between antidepressant outcomes and white matter anisotropy [25], [26]. These studies, conducted by the same group, found that individuals who failed to achieve remission exhibited lower anisotropy in multiple regions, including the anterior and posterior cingulate cortex and the dorsolateral prefrontal cortex. In contrast, we found lower likelihood of remission to be associated with increased frontal anisotropy. Despite examining smaller samples (the larger study included 48 evaluable subjects, while we examined 78), those studies had a similar 12-week study design using serotonin reuptake inhibitors and comparable demographic characteristics. One methodological difference is the use of a voxel-based analysis of the anisotropy data, while this study used a region-of-interest approach. The difference in conclusions with these studies may be related to the differences in sample size or methodology, but may also reflect heterogeneity in the pathophysiology of depression in older subjects.

This discrepancy across studies raises questions about what biological factors most strongly contribute to DTI measures. ADC is an overall measure of water diffusion, and given the size of imaging voxels relative to the microstructural environment, includes intracellular and extracellular spaces [40]. The relationship between brain microstructure and FA values are likely multifactorial. Current hypotheses of what factors contribute to anisotropy posit that myelination and axonal sheaths may restrict diffusion perpendicular to the direction of the fiber, while integrity of axonal structures such as microtubules may be associated with diffusion parallel to the fiber [41]–[43], both of which contribute to the overall FA value. Additionally, there appears to be significant heterogeneity in white matter fiber tract location and shape [44].

Another methodological difference between the studies is that our analysis method avoided hyperintense lesions. Hyperintense lesions are associated with anisotropy changes within their boundaries [19], but also are associated with widespread changes in normal appearing white matter [20]. Thus the differences in anisotropy observed in previous studies [26] may be related to clusters of hyperintensities, which would result in focal reductions in anisotropy. The same cannot be said of our current study, although the more widespread relationship between hyperintensity volume and DTI measures of normal appearing white matter [20] would be expected to be observed here.

However, hyperintensities did not likely contribute to our current findings. As greater hyperintensity severity is associated with lower FA [19], [20], this would imply that greater hyperintensity severity is associated with remission. This conclusion does not appear likely as those published studies which did find a significant relationship between hyperintensity volume and treatment outcomes have all concluded that greater hyperintensity severity is associated with lower likelihood of remission [3]–[7].

Other neuropathological differences may contribute to our findings. Reductions in FA may be related to multiple factors at the cellular level, including reduced axonal integrity or thinning myelin, although myelin may play only a modulatory role in anisotropy measures [17], [45]. Depression has been associated with deficits in neuronal size and density in frontal regions [46], [47], which would hypothetically be related to axonal integrity. This implies that older individuals who respond more favorably to antidepressants may have neuronal loss and impaired axonal integrity in frontal regions, which could result in reduced anisotropy. This leads to the hypothesis that those who experience this neuronal loss and altered neural connectivity may be more responsive to antidepressant medication, either by the effect antidepressants have on neurotransmitters or through antidepresants' neurotrophic effects. In contrast, individuals with no structural connectivity deficits may be more likely to exhibit a poor response. Functional MRI studies have demonstrated that depression is characterized by impaired functional connectivity between cortical and limbic regions, while antidepressant response is accompanied by improved functional connectivity [48], [49]. In some older individuals, this deficit in functional connectivity may be secondary to structural changes, and these individuals may respond to antidepressants better than individuals with functional connectivity deficits with intact structural connections. This is only a hypothesis, which can best be examined in the future by combining DTI and functional neuroimaging with an antidepressant trial.

One study limitation is that we could have added further covariates to the model as antidepressant remission is associated with additional factors such as disability, depression history or length of current depressive episode, and subjective social support. However, we were also limited by the sample size in the number of covariates we could include. An additional statistical concern was that we did not control for multiple comparisons, which increases the risk of a Type I error. Had we adjusted for multiple comparisons, the observed differences would not have reached statistical significance. Finally, neuroimaging limitations include that neuroimaging was not obtained until after sertraline had been started. This approach is less optimal than completing MRI before administering any drug, but one would not expect to see any changes on structural neuroimaging within a two week time frame, although such studies have not been done to confirm this. Additionally, other image analysis techniques such as voxel-based morphometry could have been used. Although this method has the advantage of examining the entire brain, it may miss critical regions of interest and does not necessarily replace a region-of-interest approach [50].

Our finding that failure to remit to antidepressant treatment is associated with higher anisotropy in the anterior cingulate cortex and superior frontal gyrus is contrary to our hypothesis and to previously published studies. However, the finding appears robust despite the study's limitations. The discrepancy across studies may reflect limitations of DTI, but may also reflect population heterogeneity – either in the depressed geriatric population, or heterogeneity in white matter structure more broadly. As such, it requires replication and emphasizes the need for larger-scale studies examining neuroanatomic correlates of antidepressant response which are linked to functional imaging studies examining functional connectivity between frontal and limbic brain regions.

## Supporting Information

Checklist S1 CONSORT Checklist
(0.06 MB DOC)

Protocol S1 Trial Protocol
(0.21 MB PDF)
