# Supplementary material for: Frontal White Matter Anisotropy and Antidepressant Remission in Late-Life Depression
Source: PLoS One. 2008 Sep 24;3(9):e3267. doi: 10.1371/journal.pone.0003267 (PMC2533397; doi:10.1371/journal.pone.0003267)
Supplement: Protocol S1 — Trial Protocol (0.21 MB PDF) [file pone.0003267.s002.pdf]

Study Design Overview

This study was designed to look at neuroimaging predictors of antidepressant treatment treatment response in older depressed subjects. In this context, we designed an open-label standardized treatment regimen to assure all subjects were treated consistently.

The manuscript we are submitting derived from two NIH-funded grants, which we attach. The R01 grant is the “parent” grant, and describes the joint Duke-Washington University project. This proposal provides detail on subject enrollment criteria and open-label sertraline dosing and clinical monitoring. The K23 grant added a diffusion tensor imaging assessment to the MRI being obtained through the parent R01 grant, but otherwise utilized similar entry criteria. What we report in this manuscript is a study examining how diffusion tensor imaging results were related to treatment outcomes with sertraline. Of note, the K23 added the diffusion tensor imaging acquisition only to the Duke site, so only Duke subjects are included.

The research design sections for each of these two grant proposals are included below.

At the editorial team’s recommendation, we also included a CONSORT checklist; however note that subjects were not randomly assigned to a study intervention. All subjects received open-label sertraline.

Study Design: Treatment Outcomes of Vascular Depression (R01)

D.1 Subjects

A total of 320 LLD subjects will be recruited from two sites, Duke University Medical Center (N=160) and Washington University Medical Center (N=160).

Recruiting Strategies and Sources

We anticipate that most of the study subjects will be recruited from current patient caseload of the investigators, the mood disorders programs at the two sites, and from referral by psychiatric, primary care, public health and geriatric medicine clinics affiliated at our centers as described more fully in the Resources section. We are committed to enrolling female and minority subjects in this trial.

Selection Criteria:

Inclusion Criteria:

1) *Age 60 or older, male or female, any race*
2) *DSM-IV criteria for MDD, assessed by SCID, unipolar, without psychotic features or acute suicidality*
3) *Minimum MADRS score $\geq$ 18*
4) *Mini Mental Status Exam score $\geq$ 21*
5) *Capacity to give informed consent and follow study procedures*
6) *English speaking*

Exclusion Criteria:

1) *MRI contraindications e.g. foreign metallic implants, pacemaker*
2) *Severe or unstable medical disorders (e.g. MI within past 3 months, end stage cancer, decompensated cardiac failure), conditions or drugs that may cause depression (e.g. systemic steroids, pancreatic cancer, uncorrected hypothyroidism), or any condition that in the investigators opinion makes the patients unsuitable for a trial (e.g. clinically significant cirrhosis or renal failure)*
3) *Any of the following diagnosed by SCID: current alcohol or substance abuse disorder, schizophrenia or other psychotic disorder, bipolar disorder, psychotic features of depression, current OCD or panic disorder. Current is within past six months. In general, subjects with a history of other Axis I disorders prior to their depression will be excluded.*
4) *Active suicidality or current suicidal risk as determined by the investigator*
5) *Significant handicaps (e.g. visual or hearing loss, mental retardation) that would interfere with neuropsychological testing or ability to follow study procedures*
6) *Known allergy or hypersensitivity to sertraline*
7) *Current episode has failed to respond to adequate trials of two prior antidepressants for at least 6 weeks at therapeutic doses.*
8) *Treatment with 50 mg sertraline or greater for at least one month in past 3 months.*
9) *Current use of psychotropic prescription or nonprescrition drugs or herbals (e.g. hypericum) except for limited use of certain hypnotics. Wash out period is described in a later section.*
10) *Mini Mental Status Exam score <21*
11) *Known primary neurological disorders including dementia, delirium, diagnosed stroke within the past 3 months, Parkinson's Disease, brain tumors, multiple sclerosis, seizure disorder.*
12) *Current psychotherapy*
13) *Cannot give informed consent*
14) *Does not speak English*
15) *Any other factor that in the investigators judgement may affect patient safety or compliance (e.g. distance greater than 100 miles from clinic)*

D.2. Considerations To Ensure Adequate Representation of Subjects with MRI Lesions

The entry criteria for age (60 years or older) as well as other selection criteria were made after reviewing our pilot data and our cumulative experience with this population. As shown by our pilot data, we believe that with these criteria we will be able to ensure adequate subjects with and without lesions and with a relatively representative range of cognitive function to test our hypotheses. However, we plan to examine rates of MRI lesions, at each site, after the initial 75 subjects and again after 150 subjects are recruited. If lesions are underrepresented, we will over- sample the old-old, late-onset depressives, and those with VRFs. In general we will attempt to recruit patients with 'real world' medical illnesses and VRFs with the exception of those listed

as exclusionary. Patients with hypertension, atherosclerosis, CAD, and diabetes will be included. This will increase the representation of patients likely to demonstrate T2H and improve generalizability. Our pilot data suggests that in "naturalistic samples" half of LLD subjects will have VRFs. Similarly, we have attempted to be inclusive with regards to those psychiatric comorbidities that would not affect our scientific rigor or patient safety. We excluded bipolar disorder since it is clearly a separate illness, as evidenced by family history, genetic and treatment response data. Actively suicidal patients are also excluded. Patients with psychotic depression will be excluded, because sertraline treatment alone is not appropriate for this group where combination antipsychotic/antidepressant or ECT is indicated. All other subgroups of patients who do meet criteria for unipolar MD, including patients with atypical depression and concomitant personality disorder, will be eligible for the study. This approach will enhance generalizability

D.3. Medication Washout

We carefully considered both sides of this issue and deliberated the pros and cons of no washout, as well as washouts shorter and longer than what we selected here. We are aware that the priority is to ensure patient wellbeing and not delay the start of treatment or discontinue treatments that were effective. We also considered a placebo lead-in allowing patients to be screened and monitored during the washout lead in period. A placebo run in was not selected because of the lack of generalizabilty to clinical practice and lack of consensus on the utility of a single blind placebo lead-in in geriatric depression. For most psychotropics, many current phase II-IV drug trials use a 2-week washout and this has generally not been a problem. We recently completed a randomized trial of elderly depressed subjects in which this issue did not pose a problem. Hence, we selected a washout period of 2 weeks. The exception is fluoxetine which requires a five week wash out. Hence, we will exclude patients currently taking fluoxetine unless they have already stopped fluoxetine for 5 weeks or longer prior to baseline. Since fluoxetine is infrequently used in the elderly, we do not expect this to be a major restriction on recruitment. ***Prior to discontinuing any psychotropic we will check with the physician who prescribed that agent, where feasible, to ensure that such a step is appropriate. During the taper/washout we will also check subject well being by appropriate monitoring. Washout will not be done in any subjects in whom it is felt to be clinically inappropriate. Patients will be counseled about symptoms of worsening/withdrawal and provided an emergency telephone contact.***

D.4. Study Design

D.4. 1. Study Duration and Schedule of Assessments

Informed consent and eligibility are determined at the Screening visit. After consent, during the initial visit patients will be screened for age, depression, ability to follow study procedures, and for MRI exclusionary criteria (e.g. pacemakers, metal) and interviewed by the study psychiatrist for medical and psychiatric history. Subjects who meet initial eligibility criteria will undergo the MADRS, mental status exam, the Structured Clinical Interview for DSM-IV (SCID) (Spitzer et al., 1992), past treatments using the Antidepressant Treatment History Form. The Framingham scale for VRFs and Cumulative Illness rating Scale for Geriatrics are also obtained at baseline. They will be screened for other significant medical and neurological disorders, dementia, alcohol and drug abuse, and other exclusions. Subjects will undergo a complete physical and neurological examination (including laboratory testing and EKG if not done in past 3 months), the Mini-Mental Status exam, and queried for all concomitant medications (see below for full description).

Upon meeting inclusion criteria, subjects will undergo MRI. Patients who are unable or unwilling to complete this procedure, e.g., claustrophobia, will not enter the trial. Before the MRI patients will be screened for claustrophobia as well as other exclusions (e.g. metal) using a check-list. Technicians will be present to reassure patients during scans. Our experience at both sites is that less than 5% of patients cannot complete the MRI scan due to claustrophobia. The scan will be archived and processed as described elsewhere. Patients with clinically significant MRI findings (e.g. tumors, large cysts) noted by the radiologist at the time of the scan will be referred for appropriate care and such patients may also be excluded from the study if the finding is likely to affect safety or confound MRI ratings. Eligible subjects will then undergo neuropsychological testing. Subjects meeting inclusion and exclusion criteria at the baseline visit will be treated with sertraline for 12 weeks. Post-baseline visits occur at weeks 2, 4, 6, 8 and 12. ***See Table in section D.5. for the complete schedule of assessments****. Telephone contacts and interim visits may be scheduled at investigator judgement if needed. A psychiatrist will see the subject at each visit. At all study visits, the MADRS, CGI and HAMD will be administered to assess the status and severity of depressive symptoms. Vital signs, adverse*

events and safety will be assessed at each visit. The SF-36, Q-LES-Q, the Duke disability assessment and neuropsychological testing are obtained both at baseline and at week 12 (or termination).

Section 3 (section following the references) includes a description of Steps to Minimize Drop Outs and Increase Retention and Handling of Drop Outs.

D. 4. 2 Rationale for Selecting a 12 Week Course of Treatment

The 12 week duration was chosen after examining all published geriatric depression trials and to allow a sufficient time for clinical response in most of this patient population (Cohn et al., 1990), including elderly patients with comorbid illness who require longer to respond, (Alexopoulos et al., 1996). In a subanalysis of a sertraline trial of patients age ≥ 70 with MD, 65% were responders by week 12 following a flexible dose titration (50-150 mg sertraline). Patients, whose initial mean 24-item HAMD score was 24, had a mean score of 12 at week 10 and had further decreased to a mean score of 9 by week 12 (Finkel et al., 1999), indicating that allowing a 12 week time course is necessary in some patients. Although there is no consensus on when to switch therapies, we have chosen to attempt to continue all patients to 12 weeks to examine the time course of response and remission rates more fully. We plan to assess subjects at every visit for safety and will be particularly vigilant in those whose depression is not responding. Subjects who develop clinically significant worsening (e.g. serious adverse events, suicidality) will be referred for immediate care and discontinued if appropriate.

D.4. 3. Choice of Sertraline as Study Drug

The favorable efficacy profile with low incidence of side effects has led to the selective serotonin reuptake inhibitors (SSRIs) becoming the most frequently prescribed class of antidepressant medications. The side effect advantages apply particularly to the elderly. As stated previously, Alexopoulos et al. have provided new insights into the effects of executive dysfunction on outcomes following nortriptyline. However, despite the wide use of the SSRIs in the elderly, it is not known if this finding is generalizable to the SSRIs. There is no firm evidence establishing superior efficacy for any one SSRI over the other available SSRIs. Sertraline has been selected as the SSRI in this study because it is among the more selective 5- HT re-uptake inhibitors, has an optimal profile for safety and effectiveness in the treatment of major depression in the context of comorbid illness (Preskorn et al., 1995; Sheline et al., 1997), has linear kinetics, has minimal age effect on clearance (Finley 1994; Nemeroff et al., 1996), and unlike paroxetine does not have anti-muscarinic effects. Although an active metabolite has been identified, the metabolite has insufficient activity to be clinically important (Finley 1994). Across the clinically relevant dose range (50-200 mg daily), sertraline does not alter its own clearance but fluoxetine and paroxetine display nonlinear dose/plasma level correlations (Finley, 1994; Nemeroff et al., 1996). Thus, adverse effects after dosage alteration are likely to be more predictable with sertraline. Sertraline causes relatively less inhibition of the hepatic P450 isoenzyme CYP2D6 compared to the other SSRIs, thereby decreasing the potential for drug-drug interactions (Nemeroff et al., 1996). Sertraline has been studied in the elderly in 4 multicenter controlled trials of which one was a placebo-controlled trial. There is also increasing pilot safety and tolerability data with sertraline in elderly patients with VRFs. Some of these differences between the SSRIs are theoretical, and practical consequences remain to be demonstrated in empirical research. Nonetheless, the evidence weighs slightly in favor of sertraline, particularly because of the relatively short half-life and potentially fewer cognitive side effects and drug interactions.

D. 4. 4. Sertraline Dosing

The initial sertraline dose will be 25 mg for one day to rule out drug sensitivity, then 50 mg daily, with subsequent dose changes at 2 weeks (to 100 mg per day), at 4 weeks (to 150 mg), and 6 weeks (to 200 mg per day) based on treatment response and side effects. *We will use CGI-S criteria to standardize this across sites. Patients with CGI-S ≥ 3 (at least mild depression) at any visit will have a specified dose increase unless there is a concern for tolerability. Patients with CGI-S of 2 (borderline symptoms) can be titrated at investigator judgment and those with CGI-S of 1 are expected to be in remission.* Given sertraline's 24 hr. $t_{1/2}$ subjects should achieve stable blood levels at each dose relatively quickly. *Stable plasma levels are achieved within 4-6 days of beginning administration in most subjects.* Some subjects (see below) who do not respond to 150 mg will respond to 200 mg, and therefore in the absence of side effects this dose will be used.

Our dosing scheme was selected after reviewing all published data in the elderly with sertraline, the package insert and our cumulative experience from six prior sertraline trials we have conducted in the elderly. This is designed to optimize response without compromising safety. While many studies have examined 50 mg of sertraline, newer evidence suggests that this dose may be too low in many patients. In a study of 1437 outpatients age ≥ 60 who were given a flexible dose titration, 48% of patients were receiving a final dose at 8

weeks of 50 mg sertraline, 36% were receiving 100 mg, 11% were receiving 150 mg and 4% were receiving 200 mg (Arranz & Ros, 1997). This study included patients with both major depression and dysthymia, and those with major depression required higher doses. The mean dose was about 90 mg in a pooled analyses of two sertraline geriatric depression trials where the dose could be titrated only up to 100 mg (study 1) or 150 mg (study 2). In a recent controlled study of 156 elderly depressives, 100 were randomized to receive sertraline whose dose could be titrated based on response to 200 mg. 72% of patients were titrated to doses of 100 mg per day or higher (Blumenthal et al. personal communication).

Section 3 (section following the references) includes a detailed plans for Obtaining and Preparing Study Medication, Drug Accountability, Dispensing, Handling Missed Doses, and Steps to Monitor and Enhance Compliance.

D.4.5. Concomittant medications/psychotherapy

Most nonpsychotropic medications will be permitted in order to enhance generalizability as long as they are not contraindicated with sertraline. We are aware of the potential for SSRI drug interactions (e.g. Krishnan, Doraiswamy, Steffens. Pocket Reference of Psychotropic Drug Interactions, 1997) and will increase our vigilance in cases where patients are on medications with such potential (e.g. beta-blockers). Patients on warfarin (coumadin) will be included after consultation with the patient's internist/cardiologist, and PT/PTT will be monitored during the study. Concomitant psychotherapy will not be permitted during the study. The reason for not including concomitant psychotherapy is the demonstration of augmentative effects on treatment outcome when used in combination with pharmacotherapy (Frank et al., 1990). A common list of key exclusionary psychoactive medications will be used by both sites. These will include other antidepressants, cholinesterase inhibitors, ginkgo, hypericum, mood stabilizers, and antipsychotics. During the trial, limited use of zolpidem or zaleplon at bedtime for sleep and lorazepam up to 1 mg daily for anxiety or agitation will be permitted. Use of hypnotics will be discouraged the night before or the day of clinical neuropsychological assessments.

D.4.6. Safety:

At each visit, all patients will be seen by a psychiatrist to assess safety and adverse events. ***Section 3 (section following the references) includes a detailed description of safety issues including definition monitoring and reporting of adverse events and serious adverse events, steps to minimize risk for suicidality and plans to handle suicidality or significant worsening, plans for emergencies and hospitalizations****, plans for handling confidentiality of subject data. Summary tables will be created for vital signs, AEs by body system using standard terminology, SAEs, drop outs and drop outs attributable to AEs. In addition to common side efefcts, we will be vigilant for rare events such as bruising, SIADH, and serotonin syndrome. In general, we expect sertraline to be relatively well tolerated.*

D.4.7. Subject Follow-up

At the end of the study, we will assist all patients to obtain ongoing treatment either through our practice, research clinic or via provision of referrals. In addition, telephone follow-up will be conducted to gather pilot data on ongoing treatment and to ensure the subjects have followed through on any referrals.

D.5. PATIENT CLINICAL ASSESSMENTS

Detailed clinical and demographic information will be obtained at initial evaluation, including gender, ethnicity, age, age at onset of depressive illness, lifetime number and type and duration of depressive episodes. History of alcohol and substance use, smoking, family history (of affective disorder, dementia, and stroke) will also be obtained. Details of these and other assessment procedures are described below.

D.5.1. Cerebrovascular risk factors (VRF)

For each subject we will rate a VRF total score using the approach of the Framingham Study (Wolf et al., 1991), which gives a validated number based on the presence and severity of VRFs, weighted according to their predictive power for stroke. The total score will be used for statistical purposes.

SCHEDULE OF EVALUATIONS Table D.5

Visit (End of Week)	Screen	Baseline	Wk 2	Wk 4	Wk 6	Wk 8	Wk 10	Wk 12*
Informed Consent	X							

Screen, History, SCID	X							
PE, EKG, Laboratory test	X							
MRI		X						
MADRS, HAMD, CGI	*X*	*X*	*X*	*X*	*X*	*X*	*X*	*X*
Psychiatrist evaluation	X	X	X	X	X	X	X	X
Vital signs, weight	X	X	X	X	X	X	X	X
AEs, SAEs		X	X	X	X	X	X	X
CIRS-G, QOL, Disability	X							X
Neuropsychological tests (*including IP)*		X						X
Concomitant Medications	X	X	X	X	X	X	X	X
Drug Dispensed		X	X	X	X	X	X	

*Wk 12 assessments will also be done if patient discontinues early

D.5.2. Psychiatric Treatment History

Exposure to psychiatric treatments in the current episode, including psychotherapy, medications, and ECT, will be obtained at initial evaluation. Necessary medical, psychiatric and other medical information will be obtained from the patient, referral source, other treating physicians, pharmacies, and hospital records. The Antidepressant Treatment History Form (ATHF), which is an expanded and modified version of prior rating scales (Keller et al., 1987; Sackeim et al., 1990; Prudic et al., 1996) will be used). The ATHF is regularly updated to include newly approved antidepressant medications. The ATHF assesses the type, dose, and duration of medication trials, and the number and type of ECT. Detailed information on these issues will be obtained for the current episode, with an abbreviated version used for prior episodes. The variables derived from the ATHF include the relative strength of medication trials and quantification of medication resistance. The ATHF will be administered to the patient at study entry.

D.5.3. Other Medication History

At every visit, all concomitant medications used will be documented in a standardized rating form. These will include all prescribed medications as well as over the counter agents.

D.5.4. Alcohol and substance use.

Social alcohol use will be recorded at entry. Patients who meet DSM-IV criteria for alcohol or other substance dependence will not be included in the study.

D.5.5. Medical Examination

At initial evaluation, all patients will undergo a complete physical examination, blood work (Chem 20, CBC, thyroid function tests, serum folate and B12, lipid panel), EKG if not done within the past 3 months. *Orthostatic blood pressure (supine to standing) and heart rate will be assessed at every visit*. Height and weight will be recorded at entry and wieght recorded at endpoint. A history of major illnesses and treatments will be taken, and the Cumulative Illness Rating Scale-Geriatric (CIRS-G) will be completed by the evaluating physician at the start of the trial (Miller et al., 1992). The CIRS-G covers all major systems (cardiovascular, pulmonary, renal, genitourinary, etc), and provides scores for current and past medical illnesses, including a global composite score that will be used as a measure of medical morbidity.

D.5.6. Assessment of Dementia

Subjects will be excluded if they have scores < 21 on the Mini-Mental Status exam or if they meet criteria (DSM-IV or NINDS) for dementia of any cause. MMSE scores below 24 are considered abnormal, however only 3% of healthy adults score below 26. We considered using a lower cut-off but chose 21 which still gives us a broader range of cognition functions. Patients meeting criteria for dementia will be excluded regardless of MMSE score.

D.5.7. Assessment of Efficacy

The *MADRS,* HAM-D and CGI will be administered at all visits in the treatment trial. Clinical efficacy evaluations include the rating scales listed below that measure the severity of the depressive symptomatology and health quality of life.

PrimaryOutcome Measure

<u>Montgomery-Asberg Depression Rating Scale (MADRS)</u>: The MADRS is a rating scale based on a clinical interview and is the instrument we used in our pilot studies. It consists of 10 items on which each item scored

on a 7-point scale, with 0 signifying the absence of the symptom and 6 signifying the most extreme form. Total score ranges from 0 to 60.

Secondary Outcomes

Hamilton Depression Rating Scale (HAM-D, 21 item): We chose this as well since it is used more widely than the MADRS in the US and in registration trials. It is intended to assess the state of the patient's condition at the time of the interview and over the preceding week. The total score ranges from 0 to 62.

Clinical Global Impression (CGI): The CGI Severity of Illness and Global Improvement are routinely used as an outcome measures in therapeutic trials. The CGI-S and CGI-I are 7-point measures, with lower scores indicating better health. The CGI-S is obtained at all visits in this trial and we plan to use this to also determine need for titration as stated under dosing. CGI-I is only obtained at post-baseline visits since it is a measure of improvement.

Quality of Life assessment: Health-related quality of life will be assessed with the Medical Outcomes Study SF-36, which requires 5-10 minutes to complete. A total score is not available but the summary scores on 8 items will be used for exploratory analyses. The Quality of Life Enjoyment and Satisfaction Questionnaire (Q-LES-Q) is a self-report measure designed to enable investigators to easily obtain sensitive measures of the degree of enjoyment and satisfaction experienced by subjects in areas of daily functioning. The summary scores have been shown to be reliable and valid measures of these dimensions in a group of depressed outpatients (Endicott et al., 1993). Both will be obtained at baseline and endpoint.

Disability assessment: We will use a 16-item self-report measure assessing two domains of physical function. These items are part of the Duke standard assessment, and have been used in our paper on disability in geriatric depression (Steffens et al., 1999b). Seven items, derived with some modification from Katz et al. (1970), Branch et al. (1984), and Nagi (1976) address physical self-maintenance skills: the ability to eat, dress, groom, ambulate, bathe, use the toilet, and bend down to pick up an object on the floor. Nine items, modified from Fillenbaum (1985) and Rosow and Breslau (1966), assess performance of IADL: getting around the neighborhood, shopping for basic necessities, preparing meals, cleaning house, doing yardwork or gardening, keeping track of money and bills, walking 1/4 mile, walking up and down one flight of stairs, and taking care of or watching children. The wording of all items followed a consistent pattern (?Can you...?), with a standardized three-option answer format [?yes? (coded 0)/ ?with difficulty? (coded 1)/?no? (coded 2)]. Composite measures were constructed for each physical function domain by summing the scores of all items within that domain. Thus, potential ranges for scores of the composite variables were 0-14 for the self-maintenance skills summary variable and 0-18 for the IADL summary variable. These composite variables are similar, but not directly comparable, to the disability section of the Philadelphia Multilevel Assessment Instrument (Lawton et al., 1982; Lawton 1988).

Safety Assessments:

Safety will be assessed in several ways including psychiatrist evaluation and vital signs at each visit, physical examination at screen, vigilance for suicidality and serious adverse events, and careful assessment and follow up of all treatment emergent adverse events till resolution. Details of assessment of AES and SAEs, worsening, suicidality, emergencies and hospitalizations are detailed in section 3.

D.5.8. Standardized Training and Quality Assurance.

Detailed description of initial training (e.g. at site and at start up meeting), rater certification standards, ongoing QC, and methods to ensure interrater reliability at an acceptable level are described in Section 3. **All clinical efficacy assessments should be done by the investigator or personnel delegated by the investigator, who is suitably trained to assess the patients.** ***All psychiatric evaluations and ratings be carried out by the same observer for a given patient, preferably in the same setting and at the same time of day. This will be monitored by assigning rater IDs.*** *Plans for back up raters to cover vacation or personnel changes are also detailed in Section 3.*

D.6. Neuropsychological Testing

The assessment will consist of well-accepted neuropsychological measures. These include the Mini-Mental State exam as a screening exam for dementia (Folstein et al., 1975), the Dementia Rating Scale (DRS) (Mattis, 1988), which has subscales for Attention, Initiation and Perseveration, Construction, Conceptual and Memory. We will be using the subscales as well as the overall score. For attention, speed of processing, and conceptualization, the Trail Making Tests Part A & B, Digit Span (forward, back; WAIS-R), and the Symbol Digit Modalities Test are included. In addition other measures as described below were designed to indicate aspects of frontal and subcortical function, which will be correlated with measures of *FDWMH* and *SCGMH*.

Test-retest reliability for the above tests have generally been reported to be ≥ 0.80.

D.6.1.Mattis Dementia Rating Scale (MDRS):

The MDRS is an easily administered screening device used in the initial diagnosis of dementia or to assess low levels of cognitive functioning (Mattis, 1976). The MDRS includes subscales that measure attention, initiation and perseveration, construction, conceptualization, and memory resulting in a total of 38 items for a maximum of 144 points. Items are hierarchically arranged so that the most difficult item is first. Adequate performance on this item allows for discontinuation of testing within that particular set (Freidl et al 1997). The Mattis Dementia Rating Scale (DRS) is particularly sensitive to changes associated with Alzheimer's Disease. Age effects are reported only for patients with moderate to severe dementia (Vitaliano et al., 1984).

D.6.1.a.Preliminary Data for IP scores in late life depression versus controls. The IP subscale of the MDRS, has been shown to be a predictor for poor or delayed antidepressant response (Kalayam et al., 1998). This subset was found to be the most predictive of functional level of "psychogeriatric" patients (Nadler et al., 1993). We will be using the IP subscale, administered according to the standardized manual (Mattis, 1988). The IP tests include 10 items worth 37 points. Subjects are asked to do the following: name supermarket items (20 points); name articles of clothing (8 points); say two sets of specific terms four times in row (2 points); palm alternations (1 point); finger tapping (alternate tap left, tap right) (1point); copy simple line drawing (1point); copy string of Xs and Os (1point).

D.6.2.Verbal Fluency: Verbal fluency, a widely used, validated test of frontal lobe function (Lezak, 1995), will be assessed by asking subjects to generate as many words as possible in a 60 second time period starting with a specific letter of the alphabet. Verbal fluency requires the subject to be able to organize thinking and develop strategies to search for words (Estes, 1974). We will use a set of letters (CFL) which has established norms as part of the Benton and Hamsher (1983) Multilingual Aphasia Exam. One point is given for each correct word with a sum for all three letter trials used for data analysis. In addition subjects will be asked to name as many words belonging to a specific category (animals) in a 60 second time period. The "Animal Category" is the easiest of the fluency formats (Monsch et al., 1992). Hart et al (1988) have found the animal naming category to distinguish Alzheimer's Disease from depression, however letter fluency did not distinguish the two groups. One point will be given for each correctly named item and a total score tabulated to be used in the data analysis. There are no significant age effects reported for verbal fluency (Spreen & Strauss 1991).

D.6.3.Stroop Color-Word Interference: This task has been shown to be sensitive to frontal lobe dysfunction (see, for example, Cohen & Servan-Schreiber, 1993), particularly the interference condition, which requires effortful, on-line processing. It is also a measure of concentration and selective attention (Lezak, 1995). A slowing in performance time with aging has consistently been found (Boone et al., 1990; Obler & Albert, 1985; Spreen & Strauss, 1991). A computerized version of the Stroop task will be used that was recently used to address changes in attentional control systems in individuals with Dementia of the Alzheimer's Type (Spieler et al., 1996). On each trial, participants will be presented with a word presented in one of four different colors (red, blue, green, and yellow). There are three major conditions: congruent, neutral and incongruent. The interference effect (incongruent minus the neutral condition) reflects the ability of the individual to control the inappropriate color name. The facilitation effect (neural minus congruent condition) provides an estimate of general facilitation from related context. The specific version of the Stroop task will measure response latencies (to the nearest ms) from the onset of the stimulus to the triggering of the voice key produced via the pronunciation of the color of the word. Any error responses will be recorded by the experimenter. We will use mean response latency per condition and mean error rates in the data analyses (as in Spieler et al., 1996). This task requires about 20 minutes to administer.

D.6.4.Wisconsin Card Sorting Test: This test was devised to study "shift of set" and problem solving ability (Berg 1948; Grant & Berg 1948). Several studies have shown that patients with frontal lobe disease have a tendency to make more perseverative errors than controls in the WCST (Grafman et al, 1990; Janowsky et al, 1989; Robinson et al, 1980). Age effects, consisting of older subjects making more perseverative errors, are generally not significant until after the age of 70 (Boone et al., 1990). In the present study, we will rely on the computerized version of the test (Heaton, 1993). On each trial participants are presented with a stimulus card at the bottom of the computer screen and asked to assign it to categories identified by four stimulus card at the top of the screen. After 10 consecutive correct responses within the same category, unknown to the participant, a new category is selected for sorting. This is repeated for six different sorts. The primary measures used are number of categories achieved (from 0 to 6), and perseverative errors (continue to sort along the wrong dimension after feedback has indicated that it was the incorrect category). The WCST is only used once to measure problem solving ability since once the pattern is solved most persons are unlikely to fail subsequent sets (Lezak, 1995).

D.6.5.Word List Memory test. This test (Morris et al., 1989) measures both short-term and long-term memory and allows for a comparison between retrieval efficiency and learning. The test will be administered using a standard protocol to present a 10 word list three times followed each time by recall of as many words as possible, then a second 10 word list with recall. Following a 30 minute delay subjects will be asked to recall the first list again. One point will be given for each recalled word during each presentation. The sum of the total number of words recalled in presentations one through three will also be calculated. Word list recognition gives the target ten word list as well as ten distractors to test simple retention.

D.6.6.Trail Making Test: This is a widely administered test of visuomotor tracking, speed, and attention (Lezak 1995). The Trail Making Test is also hypothesized to be correlated with frontal activation (Segalowitz et al., 1992). Normative studies suggest there are increasing performance times with age (Ernst et al., 1987). King et al (1993) found depressed patient's times were significantly slower in Part B. Part A: Subjects are given a sheet of paper with circles containing numbers on it. They are instructed to draw a line connecting each of the numbers in order as quickly as they can. The time required to correctly connect the circles is recorded. Part B: Subjects are given a sheet of paper with circles containing either a letter or a number. The subjects is instructed to connect the circles in order alternating between the numbers and the letters (e.g. 1-A, A-2, 2-B, B-3 ...) as quickly as they can. The time required to successfully complete this task is recorded.

D.6.7.Simple and Choice Reaction Time Measures: Simple and Choice RT: In addition to the higher level cognitive measures, we will also include two very simple reaction time tasks. These are standard tasks to look at general processing speed and have been shown to be good predictors of overall processing efficiency across a wide variety of tasks (e.g., Jenson, 1993). In the simple reaction time task, participants will simply respond to the onset of a letter. In the choice reaction time task, participants will make a discrimination between two letters before making a response. The simple reaction time provides an estimate of the speed of more input-output processes, whereas, the choice reaction time task also includes more central decision making processes. The question of interest here is whether there will be differential of peripheral and central processes in depressed individuals, and are these measures sensitive to both treatment, and severity of MRI lesions. A computerized measure of simple and choice reaction time will also be provided. On simple reaction time trials, the subject's task is to press a key on the computer keyboard whenever the letter K or B occurs. The simple reaction time response latency will be used to provide estimates of overall input and output processing, and should be relatively free of central decision operations (see, for example, Luce 1986). Because of the emphasis on motoric speed in this task and the relatively minimal attentional demands, this task should be more sensitive to basal ganglia function, as opposed to frontal functioning. The simple reaction time task will be contrasted with a choice reaction time task, in which participants will be told to press one button if a K is displayed and an alternative button if the letter B is displayed. The choice reaction time task now includes both a choice regarding which stimulus is presented along with a choice regarding which key to press. The difference between the simple reaction time task and the choice reaction time task will provide an estimate of more central choice processing speed.

This battery of tests will be administered at 0 and either at 12 weeks or termination of the sertraline trial. The anticipated time to complete this battery in elderly subjects is approximately 1-1/2 hours. While this may be taxing for some individuals, fatigue will be minimized by frequent breaks. We have found in ADRC neuropsychological testing that even subjects with dementia tolerate neuropsychological batteries up to 2 hours with frequent breaks.

D.6.8.Neuropsychological Composite Score:

A composite "frontal" score will be determined for each subject, composed of the individual Z scores from the Wisconsin Card Sort (number of categories achieved, number of perseverative errors), Stroop mean response latency and error rates, Verbal fluency score, Trails A and B score. Tests on which higher scores indicate poor performance will be adjusted so that higher scores indicate better performance on all measures for the purpose of creating a composite. This composite score will be correlated with the IP score. We have experience in this analytic technique of combining individual scores into a composite (see Kanne et al., 1998). Both the composite score and IP score will also be correlated with FDWMH in an exploratory analysis. In addition we will use the simple reaction time to correlate it with the IP score and how both of them are correlated with SCGMH to see if measures of slowing is useful in identifying basal ganglia lesions.

D.7. MRI Procedures

D.7.1. MRI Scanning Protocol: MRI studies will be performed at Washington University Medical Center using a standard protocol on a state-of-the-art 1.5T Beyond system (Siemens Medical Systems) with a UNIX based computer (Sun Microsystems), actively shielded gradients, and echo-planar capability. Data will be acquired at

Duke University Medical Center, Department of Radiology at the Center for Advanced Magnetic Resonance Development (CAMRD) on a 1.5T MRI system (GE Signa Horizon Echo-speed, GE Medical Systems, Milwaukee, WI). The data will be archived at Duke as part of the routine archiving provided as a service to users of CAMRD. At Washington University all data will be archived by Dr. Sheline's collaborators the same day of the study after transferring to the Neuroimaging Laboratory. At each center the same technician and physician will be available for all the scans to ensure that the identical technique is used. *The same researcher (MG) will conduct all of the FDWMH and SCGMH lesion severity ratings according to the modified Fazekas rating (see below) in order to minimize variability.* The same researcher (YIS) will be involved in all *T2H* volumetric measurements to minimize variability. MRI scans will be coded and all personnel involved in MRI data analysis will be unaware of clinical status.

D.7.1.a.Scanning Sequences: *1) 3D T1W acquisition: At Washington University, a 3-D fast gradient echo MR acquisition, magnetization prepared rapid gradient echo (MPRAGE) (mpr_ns_t1_4b195.wkc), will be used to generate anatomic images and at Duke the equivalent sequence type will be 3D IR-prepared Fast SPGR. Scan parameters* ***at both sites*** *are as follows: Orientation = sagittal; TR = 9.7 ms; TE = 4 ms; Inversion time (TI)= 300 ms; Excitation pulse angle = 8; FoV: 256 mm IS x 256 mm AP; matrix: 256 x 256 (x 128 slices) slice thickness = 1.25 mm; pixel size = 1 x 1 mm; voxel size = 1 x 1 x 1.25; scan time = 11.04 min. Contiguous 1.25 mm thick sagittal sections will be acquired, from which coronal slices oriented perpendicular to the anterior commissure-posterior commissure (AC-PC) plane will be generated.*
2) To evaluate for white matter hyperintensities T2 and proton density weighted scans will be obtained. At Washington University (Siemens sequence: tse7_15b_130_105b130.wkc) and Duke University (GE sequence: 2D FSE) ***the same parameters will be used****: Orientation = axial (same slice locations as the FLAIR); TR = 3300 ms; TE1(effective) = 15 ms; TE2(effective) = 105 ms; FOV = 150mm Left to right x 200 AP (rectangular FoV); 75% matrix; slices 20; pixel size = 1 x 1 mm; slice thickness =5 mm; scan time = 12 min.*
3) FLAIR sequence: This T2-weighted sequence will allow translation to the vast majority of clinical sites: At both sites the parameters are as follows: Axial acquisition; TR = 9.99 sec; TE = 105 msec; TI = 2300 msec; 20 slices; Fat saturation; 5mm slice thickness; 5 mm interleaved acquisition with no gap. Scan time = 4 min.

See Preliminary data (Section C.14) for data from NIH multi-site MRI trial that indicates compatibility of scans obtained on Siemens and GE systems*.*

D.7.2.Methods for Monitoring Image Quality

Each site will have an MR QC Phantom (MRI/QC/P, Data Spectrum, Inc, Hillsboro, NC) that consists of a water filled cylinder containing three orthogonal planes (corresponding to the x, y, z planes) with a set of precisely drilled holes on a rectangular grid spaced at 1cm intervals. This phantom will be scanned after each subject using the FSE or TSE sequence. The phantom will be analyzed by locating the center of selected holes near the edge of the phantom in each plane. The distance between the holes in each of the planes will be calculated and monitored for any changes large enough to make a significant effect on the volumes calculated. Such changes can occur if the MRI system is re-calibrated, upgraded or otherwise adjusted and often occur without notice to the users of a system. Thus the necessity for such calibration scans. The phantom scan can also be employed to perform a spatial re-normalization of the images taken by fitting a spatial transformation to the difference between previous image hole positions and current ones and then applying such a transformation to the images in question to correct the distortion.

D.7.3.Data Transfer from Duke University to Washington University:

Data will be stored at the time of acquisition on optical disk. The data will then be transferred from the MRI system to a Unix based computer workstation and the subject name and other identifying information replaced by a randomly assigned subject ID number. The data will then be placed in a Unix 'tar' file, compressed and transferred to an anonymous ftp site that is filtered to only allow access to certain IP numbers. The Washington University site will be informed of the presence of the data on the site via email. When the Washington University site computer that is allowed access has obtained the data from this site, they will email a confirmation to Duke who will then remove the data from the site. Other data such as test data, medical information or demographic information will be keyed to the random ID number and sent via mail or email between the sites.

Imaging system inhomogeneity will be removed by sequential digital filtering of the images, which will improve spatial homogeneity of signal values (Jernigan et al, 1990). Backup of the data will be stored on

separate optical disks to be stored in a geographically separate location. Data will be registered by subject code numbers to maintain blind assessment. All scans will be reviewed clinically by a neuroradiologist (Dr. Gado) to screen for clinically relevant findings. If clinically significant findings are detected, the subject will be contacted and an appropriate clinical referral will be made. Volumetric data will be analyzed on a SUN workstation using ANALYZE software.

D.7.4. T2-Weighted Hyperintensities: Subjects will receive a modified Fazekas score for frontal deep white matter hyperintensities or subcortical gray matter ratings on MRI (Krishnan et al., 1997). The Fazekas classification system provides a rough assessment of the extent of subcortical gray matter, deep white matter, and periventricular changes and provides comparability with other studies in the literature.

D.7.4.a T2H Severity Rating

All T2H will be rated blinded to treatment data.

Hyperintensities will be assessed using the modified Fazekas criteria. The modified Fazekas criteria describe MRI hyperintensities in three regions and follow an ascending degree of severity. The criteria assess periventricular hyperintensity (0=absent, 1=caps, 2=smooth halo, 3=irregular and extending into deep white matter; severe rating ≥ 3); DWMH (0=absent, 1=punctate foci, 2=beginning confluence of foci, 3=large confluent area; severe rating ≥ 3); and subcortical gray matter lesions (SCGMH)(0=absent, 1=punctate, 2=multipunctate, 3=diffuse; severe rating ≥ 2). For DWMH we will assess those occurring in frontal lobe (FDWMH)—all lesions occurring anterior to the anterior commissure, excluding the temporal lobe.

All ratings will be conducted at Washington University School of Medicine by Dr. Mokhtar Gado, a neuroradiologist with many years of experience reading clinical MRI scans and also an expert on rating white matter hyperintensities in geriatric patients with and without dementia. Dr. Gado chaired the Neuroimaging Task Force for the multi-center Consortium to Establish a Registry for Alzheimer's Disease (CERAD) and has published on T2H rating in geriatric depression. He will receive consultation from Dr. Krishnan. Data on intra-rater reliability indicates reliability for our group of > 0.9 on all MRI lesion severity ratings (Lenze et al., 1999).

D.7.4.b White Matter Hyperintensity Volume and Location

In addition, *to pursue our secondary aims,* we will use an automatic threshold method *(Lenze et al., 1999)* to determine the total volume of white matter hyperintensities. Scans will be viewed by a neuroradiologist (MG) to identify individual lesions. Lesions seen as hyperintense in both proton density and T2-weighted images will be counted; lesions that are isointense with CSF on proton density will be excluded. The area of each lesion in a particular slice will be determined using a threshold of two standard deviations above the gray-white peak. The area of each lesion will be automatically calculated by computer, (see Data), with manual editing when needed. The volume of the *T2H* for each contiguous slice containing the lesion will then be summed to yield the total *T2H* volume for the specific lesion. The total volume for all *T2H* within the two primary regions of interest, frontal deep white matter and subcortical structures, will then be calculated. Preliminary data measuring the volume of *T2H* twice for 20 subjects, blind to subject identity yielded intra-rater and inter-rater reliabilities of 0.99 each for intra-rater reliability and 0.99 for inter-rater reliability.

Hypothesis generation: In addition, to supplement this region-of-interest analysis, we will create image maps of the *T2H* that summarize the mean distribution in different patient groups. This will serve to generate new hypotheses regarding the specific regions within frontal cortex and basal ganglia that may have particular significance in predicting treatment response. *It will entail four steps. 1) identification of all T2Hs 2) coregistration of the two different MR sequences, T1-weighted and the T2-weighted image (without editing), to correct any inter-scan head motion 3) co-registering T1-weighted scan with the Talairach stereotactic atlas (Talairach and Tournoux, 1988). The combination of these two registrations allows the T2H image from the T2-weighted data to be converted into standard atlas space. 4) combining T2H images from all patients using the Statistical Parameter Mapping (SPM) software (Friston et al., 1991).* Two types of statistical parametric images will be created. By contrasting all non-responders to responders, an image showing all pixels with significant differences in regional amount of *T2Hs* between the two groups will be generated. For example, the SPM image may demonstrate that an area of pixels within the basal ganglia has significantly more patients with *T2H* from the non-responding group vs. the responding group. A second SPM processing will use the continuous response variable Δ*MADRS* for each patient to correlate the presence of *T2Hs* at each pixel. The resulting image will show where in the brain there is a significant correlation of *T2H* and treatment response. For both SPM statistical images a critical t-threshold of 2.57 or $p < 0.005$ will be used to determine significance.

D.8. Data Analysis and Hypothesis Testing

D.8.1. Data Management:

Data will be entered, managed, and analyzed at Duke by a data manager under the direction of Dr. Pieper. Data will come from several sources–standardized assessments by clinicians, personal responses by subjects, and clinical forms (e.g. MRI forms). Scannable forms can be developed and employed for a majority of the questionnaires. These forms can then be entered directly into the data files. When data are required to be entered by hand, standard protocols will be employed (double entry by separate persons, comparison and resolution of differences between entries, with final merging onto the main data file). Original forms will be mailed to Duke from Washington University with copies retained at the site of origination (original forms are necessary for the proposed Teleform software). Adverse events will be tabulated by body system and dropouts will be tabulated by reason for drop-out. In a second step, we will compare the rates of adverse events and dropout by the predictors of interest, lesion status, and executive dysfunction. The data manager will be responsible for checking accuracy and completion of the forms, and, if necessary, incomplete forms will be returned to the clinicians for completion. These forms will be stripped of personal identifiers and will retain only study identifiers to protect confidentiality. Data will be managed on UNIX based computers housed in the Aging Center. These computers have security systems in place, and are backed up nightly. Relational files will be created (in SAS or ACCESS) which will account for the replicate observations and numerous forms. From this master relational database, SAS work and analysis files will be created for the ultimate analyses proposed below.

This study has many of the attributes of the controlled clinical trial, a fixed design with specified inclusion and exclusion criteria, intervals of measurement, and outcomes. However, the agents of interest, lesion status and executive function measures, cannot be randomized, and thus there is a need for quasi-experimental analytic techniques which incorporate confounders and covariates so that threats to validity can be addressed. Our general method of analysis will use the outcome, MADRS, to first develop a model of the trajectories incorporating the confounders and covariates listed above without respect to neuropsychological measures or lesions using the methods listed above. The neuropsychological and lesion status variables will then be added simultaneously to this model to provide a final test of the specific aims. With a single test of a single outcome under a single analytic methodology, there is no requirement for Bonferroni type adjustment for multiple tests. However, as listed below, after this test, we will perform numerous follow-up sensitivity analyses which will assess the constancy of the frontal executive dysfunction or lesion effect across the covariates and site as well as look for a specificity of effect.

The primary outcome will be response to sertraline over a 12 week period as predicted by the presence of more severe MRI lesions and frontal executive dysfunction—both composite measure and IP score. Subjects with lesions are defined as those with Grade 2-3 rating for FDWMH, SCGMH, or both. Those without lesions are defined as subjects without either. As described previously, response rate for primary hypotheses is defined by changes in the MADRS score from baseline. 'Response' is to be assessed in two general ways. First, we track the outcome over time measures to define a 'trajectory' for each person. This will model the change as a smooth function (linear, quadratic, etc.). These trajectories can then be averaged and controlled analyses performed to compare groups. Second, we will model response as 'time to event' where the event of interest is remission as defined by a score less than an a priori chosen threshold value. These 2 models are detailed below.

D.8.2. Trajectory Analysis:

The outcome measure in this study (MADRS) is measured at a number of time points for each patient during the initial and continuation phases of the study (i.e., repeated measures). This permits the estimation of a time profile based on the measurements which may then be compared among various subgroups of interest. Normally, a comparison of these profiles provides a more powerful analysis of differences than does the alternative comparison of differences at a two time points—end-point analysis using only two time points--(here baseline and twelve weeks). Analyses of the repeated measures data needs to accommodate the statistical dependence among the repeated observations within subjects as well as the type of outcome. As noted above the repeated measurements can be categorical, counts or continuous. In addition, particularly in elderly populations, standard repeated measures ANOVAs are not generally useful since these methods require full response matrices. Elderly subjects may miss scheduled measurements or drop out due to illness. Thus, in the analysis outlined below we will use hierarchical linear models to create a trajectory of response; after due consideration of alternative models by both Dr. Pieper and Dr.Kraemer this was judged to be the best approach.

The analysis of change in MADRS to test ***specific aim 1a*** *will involve continuous versions of the dependent variables with measurement at replicate occasions. Since the number of observation points will vary across persons because of dropout and missed appointments, we will employ the class of models*

variously called, hierarchical linear models (HLM), (Lindley & Smith, 1972; Laird & Ware, 1982), mixed models, or random coefficient models. With this approach, each subject's repeated measures on the dependent variable are first parameterized as an individual growth trajectory plus an error term. In a second stage, the estimated trajectories are modeled as a function of differences between individuals on variables of interest (lesions, frontal executive function status, and the confounders). HLM offers several advantages over other repeated measures or cross-sectional time series models. First, HLM makes use of empirical Bayesian estimation techniques to produce optimally reliable estimates of the individual growth trajectories.(Wolfinger & O'Connell, 1993). Second, it is not necessary to assume conditional independence or compound symmetry, as HLM allows alternative and often more appropriate error structures. Third, missing data present no particular problem as estimation occurs for each respondent separately using the full information available for that respondent. Finally, time-varying covariates can be easily incorporated into the model. HLM can be estimated with PROC MIXED under SAS. For non-normally distributed dependent variables (like remission (yes/no), the SAS macro GLIMMIX can be used with PROC MIXED to estimate generalized linear mixed models. The macro uses iteratively re-weighted likelihoods to fit the model, as described in Wolfinger and O'Connell (1993).(Lindley & Smith, 1972)

Hierarchical Linear Models will be implemented as follows. Let Y_{it} represent a response variable (HAM-D) for person i (i=1,2,...,n) at time t (t=1,2,...T_i). The general form of the within-person model is as follows.

(1) $Y_{ti} = \square_{0i} + \square_{1i}t_{ti} + \square_{2i}t^2_{ti} + e_{ti}$

In model 1, t_{ti} is the time of measurement. $\square_{0i}$ is an intercept parameter representing the status of person i at baseline. $\square_{1i}$ is the linear component of the rate of change in depression for person i over time, while $\square_{2i}$ estimates the quadratic change in the rate of change (acceleration) over time, and e_{ti} is an error term which captures random variation in an individual's score(s) on the dependent variable.

The within- and between-person model for the individual change trajectories is quadratic in form, allowing for the possibility of nonlinear change in the dependent variable over time. While we will test whether a higher order polynomial is needed to fit the individual growth curves, we expect a linear or quadratic model to be sufficiently complex to fit the data. This expectation is consistent with prior applications of the HLM technique in the study of individual change(Laird & Ware, 1982; Byrk & Raudenbush, 1992). Should a linear model provide an adequate fit to the individual trajectories, model 1 will be simplified by dropping the quadratic term, and we will model linear change.

With the between-person model, variation in the $\square_{ki}$ values (the k individual growth parameters) is modeled as a function of lesions (and IP status) and other covariates as:

(2) $\square_{ki} = \square_{k0} + \square_{k1}X_{k1i} + \square_{k2}X_{k2i} \ldots + \square_{kP-1}X_{kP-1i} + U_i$

Where: k=0,1,& 2 for the intercept, linear, and quadratic components of the individual growth curves in model 2.

In model 2, there are p (p=1,...P-1 predictors and an intercept) independent variables with X_{k1} representing lesions and/or IP status, and $X_{k\,2}...X_{k\,p-1}$ representing covariates. The $\square_{kp}$ coefficients model the effects of the independent variables on: baseline differences in the dependent variable (k=0); the linear rate of change (k=1); and change in the rate of change over time (k=2). The coefficients $\square_{11}$, $\square_{21}$, and $\square_{31}$ estimate the effects of lesion severity or IP status on level of depression and change in depression over time, and provide a test of the substantive hypotheses of interest here. U_i is an error term representing between-person random variation in the dependent variable.

The within- and between-person models provide two estimates of the individual growth trajectories which can be used to correct for unreliability. In model 1, each $\square_i$ is estimated based on separate regressions for each respondent. In model 2, the $\square_i$ are estimated with a predicted score based on the X_{k1} (here, lesion severity and neuropsychological measures and the covariates). Using the error terms in each model (e_{ti} and U_{ti}), HLM uses Empirical Bayes methods to weight each estimate by its reliability and obtain composite estimators ($\square^*_{ki}$) with smaller mean square errors than either initial estimate.(Wolfinger & O'Connel, 1993; Laird & Ware, 1982; Dempster et al., 1981; Hedges & Olkin, 1985) This manner of correcting the growth curves for unreliability represents one of the unique strengths of the HLM technique. As listed above, we are aware that there are conditions and confounders which can possibly confound the relationship of frontal executive dysfunction and lesions with the depression trajectory, and we will need to control for confounders and covariates. These variables include depression clinical course of illness variables such as number of previous

depression episodes, age of onset, age of patient, gender, sertraline dose, and baseline depression severity; medical comorbidity, in particular vascular risk factors; concomitant medication; and family history of depression. Our initial models will assess the effect of the confounders without respect to the putative agents (here, lesion severity and frontal executive dysfunction), and as a final step add the 2 predictors (and their interaction) to the models. Any significant variables will be included in all subsequent analyses as a covariate. Such potentially confounding variables may in turn be useful in generating new testable hypotheses. The usual checks of assumptions in the covariance analyses will be made, including normality of the residuals, linearity, and homogeneity of variance. If the outcome data are not normally distributed or the variance heterogeneous across time, it may be possible to transform the outcome to achieve better compliance with the assumptions. Additionally, as a final check, the effect of center and center by lesion and executive dysfunction status interactions will be tested as they impact the trajectory, and, if necessary, these effects will be incorporated into the final model. Site and lesion or frontal executive dysfunction status values at baseline and their interaction will be entered into the analysis as covariates. Since all treatment decisions are done "blind" to lesion/executive dysfunction status, we do not expect to see effects at baseline (on the intercept), but hope to show that the slope of response is related to FDWMH or SCGMH values at baseline. A significant site by lesion interaction along with a significant main effect of lesion or executive dysfunction status would indicate that there is a relationship in the same direction at both sites, but stronger at one site than at the other. A significant interaction effect without a significant main effect of lesion/executive dysfunction status would indicate a relationship at one site that cancels the relationship at another, which would cast doubt on any finding. Ideally, there would be no site by lesion/executive dysfunction status effects.

D.8.3. Survival Analysis:

The models described above measure a generalized smooth trend (generally linear) for each subject and constitute our primary analysis plan. If the change (trajectory) is linear, our specific hypotheses regarding rate of change and amount of change can be addressed in a single analytic framework. However, if change is non-linear, then it is rather more difficult to assess amount of change when the outcome of interest is differences between groups in time to remission. A second class of models is often used in clinical settings where large or clinically relevant changes or defined thresholds for remission exist. Under these models, labeled survival models, time to event is the outcome of interest and rates of remission (and differences between groups in those rates) can be calculated. In the current context, 'survival' will imply survival from remission (i.e. remaining depressed). To accomplish ***specific aim 1b*** *we will employ the Cox Proportional Hazards model (Cox, 1972) and define a priori remission from the MADRS (e.g. MADRS $\leq$ 7 and not meeting syndromal criteria). This model assumes constancy of relative hazards (or risk of remission) over time, while we are interested in the relative hazards as well as changing hazards over time. We will be able to assess if the survival curves for the groups differ in shape over time by incorporating time by group interaction into the model. Addition of covariates (see above), including time-varying covariates, can easily be incorporated into this model. In addition, we are aware, that unlike standard survival models, these subjects can theoretically re-enter the risk set and remit more than once. Software has recently been developed to analyze data where multiple events can occur for a given individual (Allison, 1995). We are aware that there are other ways of defining remission. Many industry-sponsored studies define remission only at endpoint by last observation carried forward (LOCF) or completer analyses which ignore the other time points. Other studies have used criteria such as need to meet a specific threshold for a specific number of visits (e.g. HAMD<7 for 3 consecutive weeks) or need to achieve "sustained remission" (subjects who achieve and stay remitted through the rest of the trial). We chose time to remit in order to maximize the use of the information on the various time points we have collected. However, the data collected in this trial will also permit us to compare outcomes using some of these other definitions in exploratory analyses. Test of* ***specific aim 2a & b****: The measures from the frontal neuropsychological battery will be correlated with the lesion scores. The relationship between lesions and using multivariate regression, computing deriving a canonical correlation between the multiple indicators of executive dysfunction (composite and IP scores) and lesions. Since other factors are known predictors of frontal lobe measures, similar to the analytic plan for specific aims 1 above, we will develop a covariate model, and, as a final step, assess whether the canonical correlation is at all affected by covariate adjustment. We will be particularly interested in assessing if the relationship with lesions is similar across the 2 indicators or whether they are related separately. Since IP and the composite executive function scores are measured on a continuous ratio variable, as a check on the assumptions of regression, we will assess if their effect is linear or non-linear (using restricted cubic splines (Stone & Koo, 1985)). Whether linear or non-linear, we will also test the hypothesis that subjects with most severe T2H lesion scores (grades 2-3) will have the*

more impairment (lower scores) on IP and the composite frontal executive function measure than subjects with lesion scores of 0-1.

To examine secondary hypotheses, the total volume of T2H in the regions of interest-frontal deep white matter or in the subcortical gray matter can be identified separately from other areas of the brain and in a series of sensitivity analyses, we will attempt to assess if there is any particular area which is particularly important in the course of depression and treatment response.

As an exploratory hypothesis, we will test the interaction of lesions, executive dysfunction/IP status, and vascular risk factors to see if the combined effects of these variables are merely additive or are synergistic on the course of depression. To test whether there is an independent contribution from neuropsychological variables in predicting treatment response, any significant variables will be entered into multiple regression analyses with measures of T2H. These analyses will hopefully provide insight into the mechanisms for IP status, and, subsequently, into the mechanisms by which IP and lesions operate to effect differential response to sertraline Additional analyses will explore the relationship between MADRS and the cognitive measures. Finally, specific neuropsychological measures should be more correlated with the prevalence of T2Hs in specific areas. For example, the interference measures from the Stroop task, WCST, Fluency, and Trails B should be especially correlated with the degree of T2Hs in frontal areas, whereas, the overall response latency in the simple reaction time task, and overall response latency in the congruent condition in the Stroop task should be more correlated with severity of T2Hs in the basal ganglia area. Although these latter comparisons are exploratory in nature, the current authors have had success in finding neuropsychology/neuropathology relations in other populations such as Alzheimer's Disease individuals (see Kanne et al., 1998). In exploratory analyses, we will also examine change from baseline in HAMD, QOL and disability measures as well as in time to response as defined by CGI (CGI<3).

In general, we will analyze the data within an intent-to-treat framework for patients who have received at least one dose of sertraline and also analyze it only for those participants who complete the therapy as planned. This will give us a conservative estimate (intent-to-treat) and a liberal estimate (compliers and completers) of lesion severity and executive function differences. Relative to survival analysis, the trajectory analysis is rather less affected by dropout status, since all information up to the point of dropping out is incorporated into the model. In addition to response rate, we will also compare drop out rates, and tolerability as measured by TESS by body system and serious adverse events. Despite diligent efforts to retain subjects for measurement even if they cease drug use, there is the possibility that subjects may drop out of the study. If this number of dropouts is large, then we will first attempt to assess whether dropouts differed from completers on any of their demographic variables, on their response to drug to the point of dropping out, or on their baseline physiologic measures. For survival analysis, to place bounds of the effect of dropouts on the estimates, we will assess the effect of dropping out on our estimates using various models for dropping out (e.g. labeling subjects as 'not in remission' at the point of dropping out and all subsequent points). In addition, we will investigate the possible biases and impact of the dropouts on the observed results using the newer bootstrap methodology (Lavori, et al., 1995).

D.8.4 Power:

At a simplest level, this is a 2 factor design, lesion (Y/N), as it predicts treatment response. As listed in the aims, our hypotheses test main effects, and, only in secondary follow-up analyses will we attempt to assess the interaction between lesions and neuropsychological measures on depression. We show power for remission and compare the expected results with that observed in our pilot data. Power is maximized for equal distribution of the predictor. In our pilot data on CRC subjects, 63% had lesions. In our pilot sample of subjects treated with SSRIs only (n=40), 55% were without lesions. Hence, we have projected a more conservative lesion rate of about 50% which we plan to ensure by verifying lesion rates (using a rater blinded to treatment) after the initial 75 subjects are recruited. In this pilot analyses, remission rates were approximately 23% in subjects with lesions and 48% in those without lesions. Using the power tables listed in Cohen (1987) and Fleiss (1981), assuming an alpha error rate of 0.05 (two-tailed) and a beta error rate of 20% (power=80%), assuming no censoring and assuming (theoretically) a remission rate of 50%, we will be able to detect a main effects of 2.58 for any particular predictor. This odds is relatively unaffected by rates of remission other than .5. For example, for rates of remission of 0.4, 0.25, 0.15 (or their inverses), the detectable odds are, respectively, 2.64, 3.11, and 4.42.

For the continuous variables, using the power tables listed in Cohen (1987) and Fleiss (1981), assuming an alpha error rate of 0.05 (two-tailed) and a beta error rate of 20% (power=80%), at a simple level, we will correlate the trajectories of depression against the predictor of interest – lesion status. At a simple level, under this design we are able to detect a correlation of 0.16. If we assess differences in average

trajectories by group, we will be able to detect a standardized difference between the groups of 0.31 standard deviations if the groups are split 50/50 for any particular predictor, and 0.52 standard deviations if the groups are split as severely as 10/90. That is to say, if the difference in change between the groups on MADRS is 0.32 relative to the standard deviation of the change scores, we will have 80% power to declare significance. Effects of this magnitude have been labeled as 'small' to 'medium' in the power analysis literature (Cohen, 1987). In our pilot data on SSRI treated subjects, we observed a difference in the change scores for unadjusted MADRS score between lesion groups (n=40) of 5 points with a standard deviation of the change scores of 8, giving a standardized difference of up to 0.625. In adjusted analyses, the difference in MADRS was 3.4 points with a SD of change 7.

In secondary follow-up analyses, we will test main effect of lesion severity and measures from the frontal neuropsychological battery including IP status, and their interaction on outcome. For the moment, assuming an even split in sample size on the 4 cells in a 2x2 design, we will be able to detect a standardized difference of 0.62. That is to say, relative to the standard deviation of the change scores, if the difference between lesion groups at one level of IP, for example, is .62 standard units greater than the lesion difference at the other level of IP, we will have 80% chance to declare significance. Differences of this magnitude have been labeled as 'medium' to 'large' in the power literature, but are well within line given out pilot data's effects of 0.625.

Use of the sophisticated models listed above which incorporate interim points into the model should increase precision (leading to an increased power). Finally, with a sample size of 320 we should be in a position to undertake our secondary aims examining how treatment response is affected by other variables, including neuropsychological measures, disability scores, quality of life scores, cerebrovascular risk factors, age, and age of onset. We should be able to detect even moderate relationships between these variables, for example, improvement in neuropsychological measures and treatment outcome as a function of lesion volume.

6.D. RESEARCH DESIGN AND METHODS

6.D.1. General: I propose a combination cross-sectional and prospective study in depressed elders that will investigate changes in the diffusion characteristics of the dorsolateral PFC, the OFC, the genu of the corpus callosum, and the internal capsule. A sample of 120 subjects with late-life major depressive disorder aged 65 years or older will be contrasted with a sample of 120 control subjects. The control group will be matched to the study group for age and gender. The study period will extend over five years.

Subjects will be drawn from enrollees in the R01 study, *Treatment Outcomes of Vascular Depression* (M. Doraiswamy, P.I., - letter of collaboration, p 47) at Duke University Medical Center: **this will correspond with the acute phase of this proposal**. After completing this 12-week study, they will be encouraged to enter a longitudinal R01 study, *Geriatric Depression: Risk Factors for Adverse Outcomes* (D. Steffens, P.I. - letter of collaboration, p 48): **this will correspond with the longitudinal phase of this proposal**. Some control subject data will additionally be drawn from the *Conte Center for the Neuroscience of Depression*, (Ranga Krishnan, P.I. - letter of support, pp 42-43). The institutional review board has approved these parent studies; after the study protocol is explained, all subjects provide written informed consent.

6.D.2. Subjects: Depressed subjects will be those recruited for the Treatment Outcomes of Vascular Depression study at Duke Medical Center, a 12-week trial of sertraline in depressed outpatients. It is anticipated that 160 subjects will be recruited at Duke over four years, most from the patient caseloads of the study's investigators and from referral by psychiatric, primary care, public health, and geriatric medicine clinics affiliated with our centers. Control subjects will be recruited from a listing of over 1,900 community-dwelling elders from the Aging Center Subject Registry at Duke University; these individuals have expressed a willingness to participate in Duke Aging Center Research. Eligible controls have a non-focal neurological examination, no self-report of neurologic or depressive illness, and no evidence of depression. based on the Diagnostic Interview Schedule (DIS) (189) portion of the Duke Depression Evaluation Schedule (DDES) (190).

6.D.2.a. Feasibility of Recruitment: Duke University is an academically based treatment facility with an established history of recruiting depressed subjects for longitudinal studies with a neuroimaging component. Based on prior experience, the Vascular Depression Study plans to recruit approximately 40 subjects a year.

This study also has a mechanism to insure there is an acceptable rate of MRI lesions in subjects. Rates of MRI lesions will be examined after the first 75 subjects (half at Duke) and again after 150 subjects (half at Duke) are recruited. If lesions are underrepresented, we will over-sample the old-old, late-onset depressives, and those with vascular risk factors. In general, this study is designed to recruit subjects with "real-world" medical illnesses, except those listed as exclusionary.

6.D.2.b. Eligibility:

Inclusion Criteria: (1) Age 65 or older, male or female, any race, (2) DSM-IV Diagnosis of Major Depressive Disorder, (3) Minimum MADRS score $\geq$ 18, (4) Minimum Mini-Mental State Exam score > 21, and (5) Capacity to give informed consent and follow study procedures.

Exclusion Criteria: (1) MRI contraindications such as foreign metallic implants or pacemaker, (2) Severe or unstable medical disorders, conditions, or drugs that may cause depression, or any condition that in the investigators' opinion makes the patients unsuitable for a trial (e.g., clinically significant cirrhosis), (3) Any of the following diagnosed by SCID: lifetime history of alcohol or substance dependence, schizophrenia or other psychotic disorder, bipolar disorder, psychotic features of depression, dementia, or current (last six months) OCD or panic disorder. In general, subjects with a history of other Axis I disorders prior to their depression will be excluded. (4) Active suicidality or current suicidal risk as determined by the investigator, (5) Significant handicaps (e.g. visual or hearing loss, mental retardation) that would interfere with neuropsychological testing or the ability to follow study procedures, (6) Known allergy or hypersensitivity to sertraline, (7) Current episode has failed to respond to adequate trials of two prior antidepressants for at least 6 weeks at therapeutic doses, (8) Treatment with 50 mg sertraline or greater for at least one month in past 3 months, (9) Current use of psychotropic prescription or nonprescription drugs or herbals (e.g. hypericum), except for limited use of certain hypnotics, (10) Known primary neurological disorders including dementia, delirium, or diagnosed stroke within the past 3 months, Parkinson's disease, brain tumors, multiple sclerosis, seizure disorder, (11) Currently receiving psychotherapy, and (12) Any other factor that in the investigators judgment may affect patient safety or compliance (e.g. distance greater than 100 miles from clinic).

6.D.3. Acute Phase Study Design – Drawn from the parent RO1 study, *Treatment Outcomes of Vascular Depression* - *see section 6.D.7. (page 65) for schedule of assessments*

6.D.3.a. Screening: Written informed consent will be provided and eligibility determined. After consent, subjects will be screened for age, depression, ability to follow study procedures, and for MRI exclusionary criteria (e.g. pacemakers, metal). They will be interviewed by the study psychiatrist for medical and psychiatric history, focusing on current and past medication use, significant medical and neurological disorders, dementia, alcohol and drug abuse, and other exclusions; they will also receive a complete physical and neurologic examination. Subjects who meet initial eligibility criteria will be assessed with the DDES, the CGI, the Montgomery-Asberg Depression Rating Scale (MADRS) (191), the Hamilton Depression Rating Scale (HAM-D) (192), and the Structured Clinical Interview for DSM-IV (SCID) (193). If not done in the past 3 months, laboratory testing and an EKG will be obtained.
6.D.3.b. MRI: After screening, subjects will undergo cranial MRI (which includes a DTI protocol, see 6.D.9, Biological Data Collection, p 65-66). Subjects who are unable or unwilling to complete this procedure will not enter the study. Before the MRI subjects will be screened for claustrophobia as well as other exclusions (e.g. metal) using a checklist. Technicians will be present to reassure patients during scans. Duke's experience is that less than 5% of patients cannot complete the MRI scan due to claustrophobia. Patients with clinically significant MRI findings (e.g. tumors, large cysts) noted by the radiologist at the time of the scan will be referred for appropriate care and such patients may also be excluded from the study if the finding is likely to affect safety or confound MRI ratings.
6.D.3.c. Clinical Assessments: Subjects who continue to meet inclusion and exclusion criteria at the baseline visit will be treated with sertraline for 12 weeks (see dose schedule detailed below). Post-baseline visits with a psychiatrist occur at weeks 2, 4, 6, 8 and 12. Telephone contacts and interim visits will occur as needed. At all study visits, the CGI, MADRS and HAM-D will be administered to assess the status and severity of depressive symptoms. Vital signs, adverse events and safety will be assessed at each visit.

6.D.4. Acute Phase Antidepressant Treatment
Medication Washout**: There will be a 2-week psychotropic washout period before entering acute treatment. There is a priority to ensure patient well-being and not delay the start of treatment, but many current phase II-IV trials use a 2-week washout period, and a recently completed randomized trial in elderly depressed subjects at Duke had no problems with this issue. Prior to washout, we will check with the prescribing physician to assure that such a step is appropriate; washout will not be done in clinically inappropriate situations. Patients will be counseled about the symptoms of worsening or withdrawal and will be provided an emergency telephone contact number. As noted in the exclusion criteria (criterion 4 above, p 62), subjects at high risk for suicide would not be appropriate for a medication washout and so would not be included in the study.**
Antidepressant Treatment: After providing consent, completing the baseline evaluations, and completing the necessary washout, subjects will enter an acute 12-week trial of sertraline. The initial sertraline dose will be 25 mg for one day to rule out drug sensitivity, then 50 mg daily. Subsequent dose titrations at 2 weeks (to 100 mg per day), 4 weeks (to 150 mg), and 6 weeks (to 200 mg per day) are allowable and will be based on treatment response and side effects. The Clinical Global Impression-Severity (CGI-S) (194) criteria will help standardize this regimen; patients with a CGI-S $\geq$ 3 (at least mild depression) at any visit will have a specified dose increase unless there is a concern for tolerability. Patients with CGI-S of 2 (borderline symptoms) may be titrated at investigator judgment and those with CGI-S of 1 are considered to be in remission. At every visit subjects will be assessed for safety, particularly in those whose depression is not responding. Subjects who develop clinically significant worsening (e.g. serious adverse events, suicidality) will be referred for immediate care and sertraline will be discontinued if appropriate.

Nonpsychotropic medications are permitted to enhance the study's generalizability, as long as their use is not contraindicated with sertraline. Study psychiatrists will be vigilant in cases where patients are on medications with potential interactions (e.g. beta-blockers). Patients on warfarin (coumadin) will be included after consultation with the patient's internist/cardiologist, and PT/PTT will be monitored during the study. Concomitant psychotherapy will not be permitted during the study as psychotherapy in combination with pharmacotherapy augments treatment outcome (195). During the trial, limited use of zolpidem or zaleplon at bedtime for sleep and lorazepam up to 1 mg daily for anxiety or agitation will be permitted, but other psychotropics, including other antidepressants, cholinesterase inhibitors, ginkgo, hypericum, mood stabilizers, and antipsychotics, will be exclusionary (exclusion criterion 9, p 62). Use of hypnotics will be discouraged the night before or the day of clinical assessments.
Rationale for Trial Design: The 12-week duration was chosen to allow a sufficient time for clinical response in most of this patient population (196), including elderly patients with comorbid illnesses who require longer to

respond (21). A subanalysis of a sertraline trial of depressed patients over age 70 indicates that a 12-week time course is necessary to demonstrate improvement in some patients (197).

Selective serotonin reuptake inhibitors (SSRIs) are the most frequently prescribed class of antidepressant medications due to their favorable efficacy profile and low incidence of side effects. Sertraline was selected as the SSRI in the parent study because it is a relatively selective 5-HT reuptake inhibitor, has an optimal profile of safety and effectiveness in the treatment of major depression with comorbid illness (2, 198), has linear kinetics, and age has a minimal effect on its clearance (199, 200). Sertraline also causes relatively less inhibition of the hepatic P450 isoenzyme CYP2D6 compared to other SSRIs, thereby decreasing the potential for drug-drug interactions (200). **Sertraline's safety record in medically ill individuals makes it an excellent choice for this study.**

Post-Study Treatment: Upon completion of the 12-week trial, eligible subjects will be given the option to enroll in the longitudinal R01 study, *Geriatric Depression: Risk Factors for Adverse Outcomes* (see protocol below). Those who decline will be provided other referrals for appropriate treatment.

6.D.5. Longitudinal Phase Study Design – ***Drawn from the parent RO1 study, Geriatric Depression: Risk Factors for Adverse Outcomes*** *– see section 6.D.7. (page 65) for schedule of assessments*

Subjects: Subjects who complete the acute phase study will progress to the longitudinal phase; past longitudinal studies at Duke have shown good ability to acute subjects from acute trials. Elderly control subjects are recruited by this parent R01 study and paid $100 annually for their ongoing participation.

Clinical Assessments: Subjects will be seen by a geriatric psychiatrist at a minimum of every three months while euthymic. Investigators may schedule visits more frequently as clinically indicated. Situations where more frequent monitoring is indicated include, but are not limited to: patient request, suggestion or relapse or recurrence, or development of suicidal ideation. Subjects will be assessed using the CGI, MADRS, and HAM-D at each visit. Medication use will be carefully reviewed at each visit. After one year they will undergo repeat cranial MRI/DTI and a re-evaluation of the medical illness severity.

Treatment: Subjects who enter this longitudinal phase they will be followed on active treatment prescribed by geriatric psychiatrists. If sertraline was effective in the acute phase, they will continue on it throughout the longitudinal phase. They will be evaluated as clinically indicated but at least every 12 weeks for clinical response; treatment decisions are based on clinical impressions of improvement or decline, and follow an established somatic treatment algorithm (see Appendix 4 for details) (23).

The Duke somatic treatment algorithm balances flexibility with recommendations for persevering with ongoing modifications to treatment every six weeks if there is no or poor response. **At Duke, this algorithm has been shown to produce at least partial response in 87% and complete remission in 70% over one year** (23). Electroconvulsive therapy may be used in this algorithm. It is considered in cases with catatonia, active suicidal risk, physical complications to depression (e.g., nutritional), or when there is failure of at least two different classes of medication and one augmentation strategy. This decision can be made at any time.

Subjects will continue in treatment in the parent longitudinal study beyond the one-year time frame of this project. If individuals elect to exit the study at any point, appropriate clinical referrals will be provided.

Rationale for Treatment Design: This algorithm is designed to reflect "real world" treatment options, providing for the best possible care. It allows for flexibility for current practice (e.g., new treatments) and allows subjects to enter at any point in the algorithm while considering depression severity, past treatments, and individual preference. Compared to a more rigid (e.g., clinical trial) format, this approach is appropriate because it improves generalizability of findings, compensates for high recurrence rates (201, 202) that make uniform treatments impractical, is not an efficacy trial, and there is limited data on which to base a standardized treatment protocol in geriatric depression. Adequacy of antidepressant dosage will be assessed using the Composite Antidepressant Intensity Scale. The federally funded PROSPECT geriatric depression intervention trial has successfully used this instrument, which codes adequacy of dose, duration, and self-reported adherence.

Definition of Treatment Response and Relapse:

For both study phases, the MADRS will serve as the primary outcome of interest. For both phases, ***treatment response*** *will a priori be defined as MADRS<8. For the longitudinal phase,* ***depression relapse*** *will be defined as a MADRS >10. The CGI and HAM-D will serve as secondary outcome measures.*

6.D.6. Data Collection of Potential Confounders - *see 6.D.7. (page 65) for schedule of assessments*

Functional Disability Assessment: I will assess for BADL and IADL impairment using a 16-item self-report measure that has previously demonstrated disability in geriatric depression (172). Seven items, modified from Katz et al. (203), Branch et al. (204), and Nagi (205), address BADL self-maintenance skills: the ability to eat, dress, groom, ambulate, bathe, toilet, and bend to pick an object off the floor. Nine items, modified from Fillenbaum (206) and Rosow and Breslau (207), assess IADL performance: getting around the neighborhood, shopping for basic necessities, preparing meals, cleaning house, doing yard work or gardening, keeping track of finances, walking ¼ mile, walking up a flight of stairs, and taking care of or watching children. Composite measures are constructed for each physical function domain by summing the scores of all items within that domain. Potential composite scores range from 0-14 for self-maintenance skills, and 0-18 for the IADL skills. These composite variables are similar, but not directly comparable to the disability section of the Philadelphia Multilevel Assessment Instrument (208, 209). Although longer, more complete IADL assessments are available (210), this shorter and quicker form provides a thorough basic assessment.

Length of Depressive Symptoms: To control for this potential confounder, I will use two variables contained within the DDES. One is a self-report of number of previous depressive episodes; the other is a self-report of the length of the current depressive episode.

Medical Comorbidity: To consider medical comorbidity as a potential confounder, I will use the Cumulative Illness Rating Scale (CIRS) (1), modified for geriatric populations (211), to assess the burden of comorbid medical illnesses. I will examine both the cumulative CIRS total score as well as well as the vascular subscale to examine for overall comorbidity as well as risk factors specific for cerebrovascular disease.

Medications: Use of all current medications (psychotropic and non-psychotropic) will be reviewed at the screening interview and every three months throughout the study. Given that this is an intent-to-treat study, any changes in psychotropic medications will be carefully recorded. Medications affecting vascular risk factors (such as antihypertensive agents or antilipid agents) will also be recorded.

In general, medications will be classified into drug classes (e.g., beta-blockers, ACE inhibitors, SSRIs, typical antipsychotics, etc). Their effect on diffusion results will be examined in an exploratory analysis (p 67). *Given the known association between lithium and antipsychotic agents and alterations in brain structure, individuals taking these agents will be analyzed separately to determine if these agents might affect the results.*

White Matter Hyperintensity Severity: DWMH lesion volume will be measured using a semiautomated tissue segmentation technique previously been described (212) that is part of the analysis protocol for the two parent R01 projects. White matter lesion volume will be measured in each hemisphere's frontal white matter; this is a better measurement of true DWMH severity than visual rating scales, like the Coffey scale.

6.D.7. Schedule of Assessments

NOTE: these assessments are contained within the parent RO1 studies, except for the Year 1 MRI/DTI.

Assessment	Screen	Baseline	Acute Phase (12 weeks)	Longitudinal Phase (Ongoing)	Year 1
Informed consent	X			Prior to entry	
History, SCID	X				
PE, EKG, Laboratory tests	X				
Clinical assessments: includes CGI, MADRS and HAM-D	X	X	Every 2 weeks (minimum)	Every 3 months (minimum)	X
CIRS	X				X
Medication Review	X			Every 3 months (minimum)	X

Functional Disability Assessment		X			X
Cranial MRI / DTI		X			X

This assessment schedule is for depressed subjects. **Control** subjects will also sign consent, then have the same baseline and year 1 assessments, in addition to the CIRS and medication review.

6.D.8. Safety – Acute and Longitudinal Phases

At each visit, all patients will be seen by a psychiatrist to assess safety and adverse events. If a subject voices suicidal ideation at a visit where a psychiatrist is not seen (e.g., screening visit, psychological testing), a 24-hour on call psychiatrist will be contacted to evaluate the subject. The parent studies include detailed descriptions of safety issues including the monitoring and reporting of adverse and serious adverse events, steps to minimize risk for suicidality, plans to handle suicidality or significant worsening, plans for emergencies and hospitalizations, and plans for handling confidentiality of subject data. In addition to common side effects, clinical investigators will be vigilant for rare medication events such as SIADH and serotonin syndrome.

6.D.9. Biological Data Collection - Magnetic Resonance and Diffusion Tensor Imaging

Data will be acquired at the Duke University Medical Center's Center for Advanced Magnetic Resonance Development (CAMRD) on a 1.5T MRI system (GE Signa Horizon Echo-speed, GE Medical Systems, Milwaukee, WI) using the standard head (volumetric) radio-frequency coil. The data will be archived at Duke as part of the routine archiving provided as a service to users of CAMRD.

Schedule of Assessments: All subjects enrolled in the acute phase treatment will receive a cranial MRI at baseline before treatment begins. Those who agree to continue into the longitudinal phase treatment will receive another cranial MRI at the one-year follow-up period.

Preparation: All subjects will be screened by the technologist for the presence of cardiac pacemakers, neurostimulators, metallic implants, metal in the orbit, aneurysm clips, or any other circumstance where MRI is contraindicated. Any such subjects will be excluded from the study. The screened subjects will receive a preliminary interview explaining the MRI examination, and will be presented to the scanner in patient gowns to eliminate any external ferromagnetic contaminants (e.g., underwire bras, snaps, zippers, hairpins).

A standard head coil will be used. Padding will immobilize the subject's head without causing discomfort. The scanner alignment light will be used to adjust the head tilt and rotation so that the axial plane lights pass across the cantho-meatal line.

Scanning Sequences: 1) Axial 2D Proton density and T2-weighted scans will serve as a guide to the anatomy. The following parameters will be used: 24cm fov, 2mm slice, gap=0 interleave (2 acquisitions, each acquisition has slices spaced by 4mm, the second offset by 2mm from the first to fill in the gaps), Tr=4000, Te=17 (PD) and Te=100 (T2), echo-train-length=16, 256 (phase) x 256 (frequency), full bandwidth=32KHz.
2) Axial 2D Diffusion tensor imaging echo planar single shot parameters are as follows: 25cm fov, 3mm slice, gap=0 interleave (2 acquisitions, each acquisition has slices spaced by 6mm, the second offset by 3mm from the first to fill in the gaps), Tr=6000, Te=100, 128 (phase) x 128 (frequency), full bandwidth=180KHz, 6 diffusion directions each with a b-value of 1000 sec/mm^2 plus an acquisition with b=0.

Image Processing: The four diffusion data acquisitions will be averaged together after the magnitude image reconstruction in order to obtain improved signal-to-noise. The resulting improved images will be processed in each voxel to calculate the diffusion coefficients for the voxel in each of the six chosen diffusion directions. From these data the diffusion tensor eigenvalues (D1, D2, D3) and eigenvectors will be calculated (133, 213). The eigenvalues/vectors will be sorted so that D1>D2>D3. A trace image will be calculated such that the intensity in each voxel is calculated from Tr(D) = D1+D2+D3, where ADC = (Tr (D)/3). A fractional anisotropy index (FA) image will also calculated such that the intensity in each voxel is calculated from the formula below (133). FA was selected over other anisotropy values (such as relative anisotropy) because some investigators have found it has the best performance in terms of contrast-to-noise ratio as a function of signal-to-noise ratio in simulations (214).

$$FA = \sqrt{3/2} \times \frac{\sqrt{((D1-ADC)^2 + (D2-ADC)^2 + (D3-ADC)^2)}}{\sqrt{(D1^2 + D2^2 + D3^2)}}$$

6.D.10. Analysis of Imaging Data

Imaging data will be processed on a SUN workstation at NIRL, using Analyze AVW 2.5 (Mayo Foundation, Rochester, NY). Scans will be coded and no names used for confidentiality. All personnel involved in imaging data analysis will be unaware of the clinical status of subjects.

Scan Preparation: Scan images will initially be in Signa format. They will be converted to Analyze format using specialized programs developed by Dr. James MacFall that run in Matlab (version 5.3, The Mathworks, Natick, MA).

Identification of Regions of Interest: As DTI does not provide optimal resolution, T2-weighted images will serve as a guide to the anatomy. Oval regions of interest (ROIs) are a flexible means of identifying regions and will be placed bilaterally on axial images over the specific regions listed below, regardless of the presence or absence of hyperintense lesions in that region. The presence or absence of hyperintensities within the ROI will be recorded. *ROI sizes were determined by test placement of ROIs in subjects to determine optimal sizes; this was followed by consultation with local mentors*. See Appendix 3 for examples of ROI placements.

1. <u>Orbital Frontal Cortex</u>: The ROI should be placed on the most superior slice defined by the following landmarks. The posterior border is determined by the anterior appearance of the circular insular sulcus. The most superior slice may be determined in each hemisphere separately; this is defined as where the CSF of the olfactory sulcus is at least half of the OFC anterior to posterior length. Place the ROI on each side lateral to the olfactory sulcus. Each ROI should be 6x12 (area = 55mm^2) in size.
2. <u>Dorsolateral PFC</u>: Separate ROIs will be placed for the superior and middle frontal gyri bilaterally; each ROI should be 6x12 (55mm^2). The slice tested should be the lowest slice where both gyri are visible as separate structures; this may differ by hemisphere. The precentral sulcus defines the posterior boundary.
3. <u>Genu of the corpus callosum</u>: Select the lowest slice where the left and right sides of the corpus callosum are not divided. Place a 8x4 ROI (36mm^2) in the anterior corpus callosum in each hemisphere.
4. <u>Anterior limb of the Internal Capsule</u>: Place the ROIs on the most inferior slice where the anterior horns of the lateral ventricle are present. Use the anisotropy image to guide ROI placement. The ROI should be 2x12 (32.5mm^2); try not to extend into striatal tissue.
5. <u>Left Occipital lobe white matter</u>: Use the same slice as the internal capsule placement. Place a 10x10 ROI (78.2mm^2) in the left occipital white matter posterior to the lateral ventricle. Avoid DWMH if possible.

Rationale: These regions were selected because they are either 1) implicated in the pathogenesis of depression (frontal regions) or 2) may potentially contain the connecting fiber tracts of structures implicated in the pathogenesis of depression (corpus callosum, internal capsule). The corpus callosum and internal capsule measurements will be <u>experimental</u> and are not included in the primary hypotheses (pp 54-55). The left occipital lobe will serve as a control region that has not been implicated in depression.

I also considered outlining the specific regions according to anatomical boundaries rather than using spherical or oval ROIs; both larger and smaller ROI methods have limitations (215, 216). After a discussion with my mentors, I decided that a moderately sized, oval ROI was a simpler, less time-consuming method, that it could be reliable when using the anatomy to guide its placement, and that there should be no loss of sensitivity to detect diffusion changes by using this method.

Testing Reliability: All analysts will be tested for inter- and intra-rater reliability. Each analyst will measure ten scans twice with at least one week between processing sessions; at least two analysts will participate in this process at study initiation. The identifiers will be altered prior to the second processing session to assure blinding of the analysts. Acceptable intraclass correlation coefficients will be 0.8 or above. Poor reliability scores will result in review of analysis procedures, discussion with internal mentors, and retesting. Reliability testing will be ongoing to assure there is no drift in the method or interrater values; testing will be repeated every six months in the first year of data analysis, then annually.

Data Collection and Storage: No subject names will be included on any data record. Each record will include a numerical research identifier, scan date, and processing date. Each measurement in the record will include the presence or absence of hyperintensities, the slice number, the ADC and the FA. Hard copies and electronic copies of data will be stored in secured areas only accessible to research staff.

6.D.11. Statistical Analysis of Data

Acute Phase Data Analysis: Diffusion results for the ROIs will be compared between depressed and control subjects using Student's t-test. T-tests will also be used to determine the association between regional diffusion variables and clinical data, including functional impairment, length of time depressed, and antidepressant response. Response will be defined as a MADRS score <8; HAM-D and CGI scores will be secondary outcome measures. I will also test for differences between depressed subjects who are and are not medication naïve at study entry. Linear regression models will be used to control for age, gender, medical illness severity as measured by the CIRS, and use of medications affecting cerebrovascular risk factors.

Longitudinal Phase Data Analysis: Using t-tests, 1-year scan results will be compared between depressed and control subjects; within each group, they will also be compared to the baseline scan results to determine if one group exhibits a greater rate of change than the other. A similar analysis will be performed comparing depressed subjects who 1) do and do not experience symptom remission, and 2) depressed subjects who do and do not relapse over the one year period. Linear regression models will control for age, gender, CIRS, and use of medications affecting cerebrovascular risk factors.

Exploratory Analysis - Psychotropic Use: I will also examine the effects of psychotropic medications. Past studies examining the effect of medications on brain structure have used treatment periods from 4 weeks (176, 177) to 24 weeks or over a year (179, 182, 183). Rather than select an arbitrary time frame, I will incorporate a factor measuring time by exposure to drug; this will be done for individual agent classes (such as SSRIs, TCAs, etc). Individuals on agents thought to alter brain structure (antipsychotic agents and lithium) will be analyzed separately.

Power Analysis: Power was estimated assuming a two level case/control design. As outcome measures were continuous, power was calculated using Student's t test to determine the least detectable difference from control levels. The primary interest was in the least detectable difference (decrease) in depressed subjects scores from control levels. Control levels and associated standard deviations were estimated from preliminary data of anisotropy results in frontal white matter; anisotropy was used as it is thought to be a sensitive measure of white matter tract integrity (136, 137). For anisotropy readings, I would expect to detect decreases of approximately 11% to 14% four times out of five (mean control=350.0, mean case=301.4, standard deviation=95.0, least detectable difference=48.6, %change=13.90%). With 120 depressed and 120 control subjects at both study phases, my ability (power) to detect even small differences is large (> 0.9) in the analyses of depressed and control subjects. The detectable differences described here are also well below the differences seen in anisotropy of T2 hyperintensities when compared with normal-appearing tissue.

6.D.12. Experimental Data Analysis Techniques

Neuroimaging in general and DTI in particular is a rapidly evolving field. Research is focusing not only on improving our imaging capabilities, but on improving our methods of analyzing data. **To meet my career goals of becoming knowledgeable of image data analysis techniques**, I will learn about 1) statistical parametric mapping (SPM) and 2) fiber tract mapping. As part of this learning process, I plan to apply these techniques to data I will collect as part of this study. **As these two techniques are highly experimental and their full utility is not completely understood, I am not including them as primary methods for testing my specific aims and hypotheses** (pp 54-55).

SPM: SPM can provide a voxel-by-voxel association between imaging and clinical data (217). Although initially developed as a means for analyzing functional imaging data, it has been shown to be applicable in structural imaging and in psychiatric disorders (34). SPM will be performed on a SUN workstation using SPM99 (Wellcome Department of Cognitive Neurology). The anisotropy and diffusivity maps will be normalized in SPM using parameters determined by a normalization of the image with no diffusion weighting (the b = 0 image). Each normalized image can then be smoothed with a low-pass three-dimensional spatial filter with a kernel value of five pixels in the x, y, and z directions (218). Normalized images can then be coregistered.

These data can then be analyzed in a variety of means. Two group t-tests can examine regional differences in anisotropy and diffusivity between depressed and control subjects as well as differences between antidepressant responders and nonresponders. SPM has been previously used with DTI data in epilepsy (218-220) and schizophrenia (221).
